# llluminating the live-cell dynamics of early interactions between neutrophils and the microsporidian parasite *Encephalitozoon cuniculi*

**DOI:** 10.1186/s12866-026-04989-7

**Published:** 2026-05-11

**Authors:** Eugénie Carriere, Enzo Julien, Juliette Gilbert, Julien Verlaguet, Marjolaine Vareille-Delarbre, Frédéric Delbac, Pascale Gueirard

**Affiliations:** 1 CNRS UMR 6023, Laboratoire Microorganismes : Génome et environnement, Université Clermont Auvergne, Clermont-Ferrand, F-63000 France; 2 Laboratoire Microorganismes : Génome et Environnement , 1 Impasse Amélie Murat , Aubière Cedex , TSA 60026 - CS 60026 - 63178 France

**Keywords:** Microsporidia, Polymorphonuclear leukocyte, Innate immunity, Live-cell imaging, Murine model

## Abstract

**Background:**

*Encephalitozoon cuniculi* is an obligate intracellular parasite that affects birds and mammals, including humans, and belongs to the phylum Microsporidia. The innate immune system serves as the first line of defense against infection, but it only partially clears the parasite. Microsporidia are known to modulate the effector functions of phagocytes. They can survive and multiply inside macrophages (MOs) to evade destruction after phagocytosis and also modify the functional properties of dendritic cells. However, the role of neutrophils (PMNs) in microsporidiosis remains poorly understood. In a mouse ear skin model, inflammatory cells, including MOs and PMNs, were rapidly recruited to the cutaneous injection site, and intact spores persisted within phagocytes for several days. We hypothesized that Microsporidia also manipulate the antimicrobial mechanisms of PMNs, including phagocytosis and the formation of neutrophil extracellular traps (NETs), to enhance their survival.

**Results:**

Using live and static cell imaging in vitro, along with the mouse ear pinna model, we investigated the dynamics of interactions between PMNs and *E. cuniculi* parasites. Our observations revealed that the parasites rapidly adhere to and are phagocytosed by PMNs. After activation, infected PMNs exhibited modified structural and functional properties, such as the extension of pseudopodia, parasite lysis inside phagolysosomes, modified motility and shape parameters, and NETs formation. Interestingly, a small proportion of the parasites survived intracellularly, with early developmental stages (meronts, sporonts) observed within PMNs at 24 and 48 h post-infection. Importantly, infected PMNs harboring intact and infectious intracellular spores were later phagocytosed by MOs.

**Conclusion:**

Using innovative microscopy and imaging approaches, we discovered that *E. cuniculi* Microsporidia manipulate PMNs, key innate immune cells, by using them as intracellular niches for their development, and as potential vehicles for their silent transfer to other host target cells.

**Supplementary Information:**

The online version contains supplementary material available at 10.1186/s12866-026-04989-7.

## Background

*Encephalitozoon spp*. are eukaryotic, unicellular, spore-forming microorganisms that belong to the phylum Microsporidia. They are obligate intracellular parasites of birds and mammals, including humans, and can induce multi-organ pathogenesis. Infections typically occur through the gastrointestinal tract and can range from asymptomatic to serious diseases, with some cases resulting in mortality. In immunocompromised hosts, the infection can progress to disseminated and fatal disease, while in immunocompetent mammals, it often remains chronic and asymptomatic.

The highly resistant spore is the only extracellular microsporidial form that is the infective stage [1]. Microsporidian spores contain a specialized invasion apparatus, the polar tube, which is rapidly extruded upon stimulation and functions as a syringe-like structure to inject the infectious sporoplasm into host cells. Once inside the host cell, the sporoplasm of ungerminated spores can be delivered intracellularly after subsequent polar tube discharge (spore germination). Other host cell invasion mechanisms have been described, as the sporoplasm can also be directly delivered into the host cytoplasm through membrane piercing or can be internalized by endocytosis after deposition at the cell surface [2]. The sporoplasm develops into meronts during merogony. Meronts then differentiate into sporonts, which undergo sporogony to form sporoblasts that mature into resistant spores. These spores are released after host cell rupture and can infect new cells [3].

In response to infection, the innate immune system serves as the first line of defense, but only allows partial clearance of the parasite [4]. Chronic inflammation and persistent high antibody titers are commonly observed in infected humans [5–9]. In the mouse ear pinna model, inflammatory cells, including macrophages (MOs) and neutrophils (PMNs), are rapidly recruited to the cutaneous injection site, but intact spores persist within phagocytes for several days [10]. The host immune system’s inefficiency to fully eliminate the parasite is mainly due to *Encephalitozoon spp*’s ability to modulate the effector functions of MOs and dendritic cells (DCs) to avoid destruction [1]. These hacked innate immune cells can then serve as “Trojan horses”. During disseminated disease, infiltrates of microsporidian-infected inflammatory cells, such as MOs, are detected in lesions, microabscesses, and granulomas, at distance of the inoculation site [11]. These cells therefore contribute to parasite dissemination throughout the whole host body [11–13].

We hypothesized that *Encephalitozoon spp*. could also manipulate the antimicrobial mechanisms of PMNs. These key innate immune cells possess strong antimicrobial properties. They rapidly migrate to the infectious sites to engulf and eliminate microorganisms, while also producing cytokines and chemokines. At the same time, they release antimicrobial factors and neutrophil extracellular traps (NETs) into the extracellular milieu, two processes that contribute to the killing of microorganisms before their complete engulfment. Within phagolysosomes, microorganisms are rapidly killed and degraded after being exposed to PMNs enzymes, antimicrobial peptides, and reactive oxygen species (ROS). Finally, PMNs products (cytokines, ROS, and NETs) help modulate both innate and adaptive host immune responses [14–16].

To which extent PMNs contribute to the *Encephalitozoon spp*. infection outcome remains elusive. To gain insights in the PMNs and *Encephalitozoon cuniculi (E. cuniculi)* interplay, we used live and static cell imaging to explore the dynamics of interactions between murine PMNs and *E. cuniculi *in vitro*.* We first observed that some parasites rapidly adhered to PMNs. We also bring evidence of specific changes in the structural and functional properties of PMNs that support immune activation. Importantly, despite the impressive antimicrobial arsenal of PMNs, a small proportion of viable intracellular parasites were detected, initiating development within these otherwise short-lived phagocytic leukocytes. Dying PMNs harboring viable intracellular parasites were subsequently exploited by *E. cuniculi* as potential “shuttles” to infect silently MOs.

## Materials and methods

### Mice and ethics statement

LysM-EGFP transgenic mice (8- to 12-week-old males and females) (EGFP^+^ fluorescent phagocytes) were obtained from the Biology and Genetics of Bacterial Cell Wall unit at the Pasteur Institute (Paris, France), while C57BL6 mice (8- to 12-week-old males and females) were acquired from Charles River. Both mouse strains were bred in the animal facility at Université Clermont Auvergne (Clermont-Ferrand, France). Infected mice were housed in ventilated plastic cages. All experiments were approved by the Ethics Committee on Animal Experimentation of Auvergne (C2E2A), Clermont-Ferrand, France (approval number: 28868) and conducted in accordance with relevant guidelines and regulations. To design our experiments, male and female mice of the different generations of animals (F1 to F5) were randomly chosen. To purify PMNs from the bone marrow or from ear infected tissues, mice were first anesthetized by intraperitoneal injection of a mixture of ketamine (50 mg/kg) and xylazine (5 mg/kg), and further euthanized by cervical dislocation.

### *Encephalitozoon cuniculi* spore production

*E. cuniculi* (GB-M, genotype I) parasites were propagated in HFF (Human Foreskin Fibroblasts) cells (HFF-1 SCRC-1041 ATCC cell line) maintained at 37 °C in Minimum Essential Medium (MEM) supplemented with 10% Fetal Calf Serum (FBS) and 1% L-Glutamine in a 5% CO₂ atmosphere [10]. Spores were collected from the supernatant of infected cells, washed twice, and resuspended in PBS buffer after two rounds of centrifugation (5 min at 550 x g). The number of residual HFF cells and/or cellular debris in the spore preparation was assessed by staining with 0.025% Trypan Blue solution (Gibco).

### Fluorescent labeling of *Encephalitozoon cuniculi *spores and inoculum properties

Purified *E. cuniculi* spores were stained with a 0.01% CW (CalcoFluor white) M2R solution (Sigma Aldrich) in PBS at room temperature (RT) for 2 min. After washing with ultra-pure water, CW^+^ labeled-*E. cuniculi* spores were centrifuged for 5 min 550 x g resuspended in PBS, and immediately used for the infectivity assay at MOI 50 on HFF cells. Three days pi, the presence of infectious foci was assessed using the FISH method [10, 17]. To check the labeling effectiveness, smears containing 5 × 10⁵ CW⁺-labeled *E. cuniculi* spores were made on coverslips following inoculum preparation. After overnight drying, methanol was added, and the coverslips were incubated at − 80 °C for 15 min, then rinsed with 1× PBS. Samples were mounted using ProLong Diamond Antifade Mountant (Molecular Probes) and imaged with a 100× objective using a ZEISS Axio Imager Apotome microscope. Labeling was homogenous, as all spores of the inoculum were labeled and brightly fluorescent (Additional file 1: Figure S1A). In parallel, ultrastructure of ten million of *E. cuniculi* CW-labeled spores was analysed by scanning electron microscopy (SEM), as previously described [10]. Additional file 1: Figure S1B illustrates a characteristic ellipsoid or more rounded morphology, with a smooth and homogeneous surface (Additional file 1: Figure S1B).

### Infection of HFF cells by *Encephalitozoon cuniculi*

HFF cells were distributed on coverslips in 24-well cell culture plates (8 × 10^4^ cells/well) and incubated overnight, as previously described [10]. Purified CW^+^ labeled-*E. cuniculi* spores were then added to the cell cultures (MOI 5). At 1, 4, 18, 24, and 48 h pi, the infected cell coverslips were fixed with 4% PFA (paraformaldehyde) and stored in PBS at 4°C.

### Infection of J774 cells *by Encephalitozoon cuniculi*

J774 murine macrophages (J774A.1 TIB-67 ATCC cell line) were cultured in 24-well plates containing glass coverslips at a concentration of 1 × 10⁵ cells per well in RPMI medium supplemented with 10% FBS and incubated at 37 °C with 5% CO₂. Cells were either infected or not (control cells) with *E. cuniculi* CW⁺ spores at MOI 5, and incubated for 1 h at 37 °C with 5% CO₂. After 1 h of incubation, cells were washed three times with RPMI 10% medium and incubated with fresh medium for 4 days. At each time point, coverslips were washed once with PBS, fixed with 4% PFA for 15 min at RT in the dark, and then stored in PBS at 4 °C.

### Purification of murine PMNs

Murine PMNs were isolated from the bone marrow of LysM-EGFP or C57Bl6 mice, as previously described [18–20]. A detailed description of the purification protocol is given in supplementary Materials and methods section. The purity of viable CD11b^+^ cells was 97.1% ± 0.8%, and within the CD11b^+^ population, the purity of PMNs cell suspension (CD11b^+^Ly6G^+^ double positive cells) was 91.4% ± 1.8% (Additional file 2: Figure S2A). Cell viability of the PMN cell suspension was high, with only 1,7 *±* 1.6% of dead cells (*n* = 20) (Additional file 2: Figure S2A). May-Grünwald-Giemsa (MGG) staining was performed to analyze the morphology of purified PMNs. Acquisitions were realized with a 100x objective on a microscope equipped with a Moticam S3 camera. Purified mature PMNs displayed a characteristic multilobed nucleus (Additional file 2: Figure S2B).

### In vitro PMNs infection

Purified PMNs were distributed in 24-well cell culture plates (5 × 10^5^ cells/well) and cultured at 37 °C in RPMI supplemented with 10% FCS and 1% L-glutamine, 15 mM HEPES, and 1 mM Pyruvate Sodium in a 5% CO₂ atmosphere. *E. cuniculi* CW^+^ spores were added to the PMN cultures at an MOI 5. The cells were centrifuged at 20 x g for 5 min and incubated at 37 °C. At different time points after contact (1, 4, 18, 24, and 48 h), infected cells were collected, centrifuged (450 x g, 5 min at 4 °C), and resuspended in a buffer (PBS, pH 7.4-EDTA 2 mM –BSA 0.5%). The culture supernatants of infected and uninfected PMNs were frozen and stored at -80 °C until cytokine analysis was performed.

### Immunolabelings of HFF, PMNs or J774 cells infected in vitro

#### HFF cells

A double immunolabeling was performed to quantify the proportion of adherent, intracellular, and developing parasites within HFF cells. A detailed description of the immunolabeling protocol is given in supplementary Methods and Protocols section.

#### PMNs

A specific immunolabeling was performed on collected PMNs to quantify the proportion of adherent and intracellular parasites. A detailed description of the immunolabeling protocol is given in supplementary Material and methods section.

#### J774 cells

Immunolabelings were performed on J774 infected cells to analyze parasite development inside these cells. A detailed description of the immunolabeling protocol is given in supplementary Material and methods section.

### Immunolabelings of ear tissue cryosections from *E. cuniculi*-infected mice

Immunolabeling was performed on ear tissue cryosections from infected mice, collected between 2 h and 7 days pi, or post-inoculation of PBS. A detailed description of the immunolabeling protocol is given in supplementary Material and methods section.

### Immunolabelings of PMNs purified from cutaneous *E. cuniculi*-infected tissues

PMNs were purified from ear tissues of mice infected for 48 h, as previously described [21]. Briefly, ear tissue samples were dissociated and incubated for 1 h in RPMI 10% cell culture medium, supplemented with 50 µL of DNase (5 mg/mL) and 500 µL of collagenase type IV (4000 U/mL). After digestion, the tissue sample was filtered through a 40 μm sieve to remove white connective tissue. PMNs were further isolated from the cell suspension using a mouse purified Neutrophil Isolation Kit (Miltenyi Biotec). Purified cells were then centrifuged on a CytoSpin (Shandon) at 12 x g for 5 min. The resulting cell spots were fixed with 4% PFA for 5 min and stored on glass slides at -20 °C until use. Specific immunolabelings were further performed to detect parasites inside PMNs. A detailed description of the immunolabeling protocol is given in supplementary Material and methods section.

### Transmission electron microscopy of infected PMNs

At one- and two-days pi, PMNs were collected, and Ly6G^+^CW^+^ living infected PMNs were sorted using a FACS (Fluorescence-activated cell sorting) Aria Fusion SORP cell sorter. The sorted cells were fixed overnight at + 4 °C and further processed for TEM analysis. A detailed description of the protocol is given in supplementary Material and methods section.

### Assessment of PMN activation

PMNs were distributed in 24-well cell culture plates and harvested at 1, 4, 18, 24, and 48 h after exposure to CW^+^ *E. cuniculi* spores. Two control conditions were tested: uninfected cells and cells treated with *E. coli* LPS (Lipopolysaccharide) (100 µg/mL), Thermo Fisher Scientific). LPS was added for 30 min at 37 °C under 5% CO_2_ before sampling. At each time point, cells were labeled for 25 min and incubated on ice with anti-CD11b (PE, Clone M1/70.15.11.5, dilution 1:100 and anti-Ly-6G-APC-Vio770 REA526, 0.3 µg/mL, Miltenyi Biotec) monoclonal antibody. Cell viability was measured using Sytox Green (Molecular Probes). Samples were analyzed on a LSR FORTESSA X-20 (BD Biosciences). CD11b expression levels were measured within the Ly6G^+^ PMNs population, after excluding debris, doublets, and dead cells.

### Detection of NETs by immunolabeling

PMNs were distributed into 4-well Lab-Tek II Chamber Slides (5.2 × 10^5^ cells/700 µL). Three conditions were tested: uninfected cells, cells supplemented with 150nM/ml of phorbol-12-myristate-13-acetate (PMA) (Sigma-Aldrich) for 3 h (positive control) and PMNs infected with CW^+^ *E. cuniculi* spores for 6 h. PMA was used to activate the phagocytic properties of PMNs and to induce the formation of NETs. At 6 h pi, cells were fixed with 4% PFA and stored in PBS at 4 °C. Specific antibodies were applied to detect the formation of NETs: a primary rabbit monoclonal [EPR17785] anti-Histone H3 antibody (2.66 µg/mL PBS-BSA 3%-Triton 0,01%, ABCAM) incubated overnight (OV) at 4 °C, followed by a goat Anti-Rabbit IgG H&L, TRITC (5µg/mL, PBS-BSA 1%-Triton 0,01%) for 1 h at RT, or a primary rabbit monoclonal anti-Elastase antibody (0.25 µg/mL PBS-BSA 3%-Triton 0,01%, ABCAM) incubated for 1 h at RT, followed by a goat Anti-Rabbit IgG H&L AF488 (5µg/mL, PBS-BSA 3%-Triton 0,01%) for 1 h at RT, or a rabbit recombinant multiclonal antibody [RM1001 (ab281584)] anti-Histone citrunillated H3 antibody (0.33 µg/mL PBS-BSA 3%-Triton 0,01%, ABCAM) incubated for 3 h at RT, followed by a goat anti-Rabbit IgG H&L AF488 (5µg/mL, PBS-BSA 3%-Triton 0,01%) for 1 h at RT. All samples were counterstained with DAPI (0.4 mg/L Sigma Aldrich) and mounted using ProLong Diamond Antifade Mountant (Thermo Fisher Scientific). Images were randomly acquired by confocal microscopy. NETs quantification was performed on confocal acquired images that covered half of the surface cell culture wells, based on DAPI staining, as labeling intensity was higher with this marker compared to elastase labeling. PMNs nuclei were detected (DAPI channel) using Cellpose software (base parameters + Requested pixel size = 0, Minimum area = 15µm^2^). 5000 cells were randomly selected using a script and manually assigned a class (NETs or ignore). Cells emiting DAPI + DNA fibers were considered as positive for NETs formation. Negative cells exhibited a typical DAPI+ polylobed nucleus with absence of DNA+ emiting fibers. Results were expressed as percentages of cells positive for NETs formation for the three experimental conditions: uninfected PMNs (NI), positive control PMA (PMA) and infected PMNs (I).

### Video microscopic imaging and data acquisition

PMNs purified from LysM-EGFP transgenic mice were distributed in µ-dishes containing a 2-well or a 4-well micro-Insert (Ibidi), at a density of 8 × 10^4^ and 1.3 × 10^5^ cells per well, respectively. Two conditions were tested: uninfected PMNs and *E. cuniculi*-infected PMNs (MOI 10). Time-lapse acquisitions were performed on a ZEISS Spinning Disk Cell Observer (SD) (Carl Zeiss Microscopy) or a ZEISS LSM 980 confocal microscope (Carl Zeiss Microscopy) using a 40x objective, with imaging conducted for 2 h.

### Video microscopic imaging and data analysis

Randomly acquired videos were analyzed using Imaris software to detect both cells and parasites. The detailed data analysis protocol is given in supplementary Material and methods section. The motility parameters (mean speed, displacement length and trajectory straightness) along with morphological characteristics (area, volume and prolate ellipticity) were compared between uninfected, and infected PMNs in contact with spores, over the 6 h of incubation time. For ellipticity prolate parameter, additional analyses were performed to compare values between the uninfected and infected PMNs at three distinct time points: early (< 1 h), intermediate (2–5 h), and late (5–6 h).

### Co-incubation of MOs or HFF cells with *E. cuniculi*-infected PMNs

J774 cells were cultured on 8-well LAMETECK chamber slides whereas HFF cells were cultured on 24-well cell culture plates (1.12 × 10^5^ cells/300 µL or 8.10^4^ cell/500µL respectively for J774 or HFF cell lines, Lab-Tek II, Thermo Scientific).

*E. cuniculi*-infected sorted PMNs (18 h pi) were co-incubated with MOs (ratio 10 :1) or HFF cells (ratio 5 :1) for 1.5 h. After contact, J774 or HFF cells were rinsed with RPMI 10% and incubated in cell culture medium for respectively 18 h (J774 cells) or 72 and 96 h (HFF cells). Immunolabelings were further performed on J774 cells whereas FISH was performed on HFF infected cells.

### Immunolabelings of J774 cells co-incubated with *E. cuniculi*-infected PMNs

J774 infected cells were further fixed with 4% PFA for 15 min, and specific immunolabeling was performed. A first saturation step was realized using PBS-BSA 1%, followed by incubation with a primary antibody: anti F4/80 Monoclonal Antibody BM8 (5 µg/mL in PBS-BSA 1%-Triton 0.01%, Fisher Scientific), overnight at 4 °C (eBioscience) coupled with an anti-rat-AlexaFluor 647 secondary antibody (2 µg/mL in PBS-BSA 1%-Triton 0.01%, Thermo Fisher Scientific) for 1 h. A second saturation step was performed, and two additional antibodies were applied: a polyclonal *E. cuniculi*-antiserum (2 µg/mL in PBS-BSA 1%-Triton 0,01%) coupled with an anti-rabbit IgG-AlexaFluor 488 secondary antibody (2 µg/mL in PBS-BSA 1%-Triton 0,01%, Thermo Fisher Scientific) for 1 h and an anti-NIMPR14 antibody (sc-59338) (Santa Cruz Biotechnology) (1 µg/mL, PBS-BSA 1%-Triton 0.01%) during 2 h, which was coupled with an anti-rat-AlexaFluor 546 secondary antibody (4 µg/mL, Thermo Fisher Scientific, PBS-BSA 1%-Triton 0.01%, for 2 h). All samples were counterstained with DAPI (0.4 mg/L, Sigma Aldrich) and mounted with ProLong Diamond Antifade Mountant (Thermo Fisher Scientific).

### Imaging the parasite development using Fluorescence *in situ* hybridization

FISH was performed on infected and uninfected PMNs purified from the bone marrow or from the ear tissue of infected mice, on J774 infected cells or on HFF cells co-incubated with *E. cuniculi*-infected sorted PMNs in vitro. Labeling was followed by counterstaining with DAPI and Direct Yellow (DY96) 1 µM. A probe targeting the *E. cuniculi* rRNA (Ec01, 5′-CCACAGGGGCAGACCACTAT-3′) was used, as previously described [10, 17]. FISH labeling enabled the detection of all Microsporidia stages, except spores. The combination of DAPI (nucleic acid stain) and DY96 (chitin stain) allowed visualization of infection foci, regardless of the parasite stages present (sporoblasm/meronts/sporonts/ sporoblasts: FISH; sporoblasts: FISH and DY96; sporoblasts/spores: DY96). A detailed description of the FISH protocol is given in supplementary Material and methods section. Coverslips were mounted with Prolong Diamond (Thermo Fisher Scientific) and images were acquired on a ZEISS Axio Imager microscope (Carl Zeiss Microscopy, Germany) with a 63x objective. Other acquisitions (20 to 30 random fields per coverslip of infected PMNs or HFF) were performed on a Spinning Disk microscope (40x objective) to quantify intracellular developing parasites numbers. Using Fiji (ImageJ) software, the proportion of Cy3.5-labeled probe+ parasites (red fluorescence signal) was calculated, normalized to PMNs nuclei numbers and expressed as a percentage.

### Acquisition of fluorescence microscopy images

Images of FISH and immunolabelings performed on HFF cells, J774 cells and purified PMNs were first acquired using a ZEISS Axio Imager microscope (Carl Zeiss Microscopy, Germany) with a 63x (oil) objective. Additional images were randomly acquired on a ZEISS LSM 980 confocal microscope (Carl Zeiss Microscopy, Germany) equipped with an Airy Scan module and observed with a 63x (oil) objective. Images of immunolabelings performed on ear tissue cryosections and on purified PMNs infected in vitro (NETs quantification) were acquired using a ZEISS LSM 800 confocal microscope (Carl Zeiss Microscopy, Germany) with 20x and 40x objectives (CLIC platform, Faculty of Pharmacy, UCA).

### Statistical analysis

Data were analyzed using a Mann-Whitney non-parametric one-tailed test on Prism 8 or 10 software (GraphPad Software, Inc). NETs vs. no NETs’ binary data, obtained in each of the three groups (PMNs, PMA and PMNs + E.c) were compared with a binomial GLMM (glmer function of the lme4 package) in R [R version 4.5.1 and R studio (Version 2025.09.1 + 401), assisted by ChatGPT (custom GPT, model GPT-5 Thinking, accessed October 2025). EMMeans and Tukey comparisons were performed using the emmeans package. A p-value ≤ 0.05 was considered statistically significant, with the following significance levels: *p* < 0.05 (*), *p* < 0.005 (**), *p* < 0.001(***) and* p* < 0.0001(****); ns = not significant).

## Results

### Dynamics of global interactions between PMNs and *E. cuniculi* parasites

**Fig. 1:**
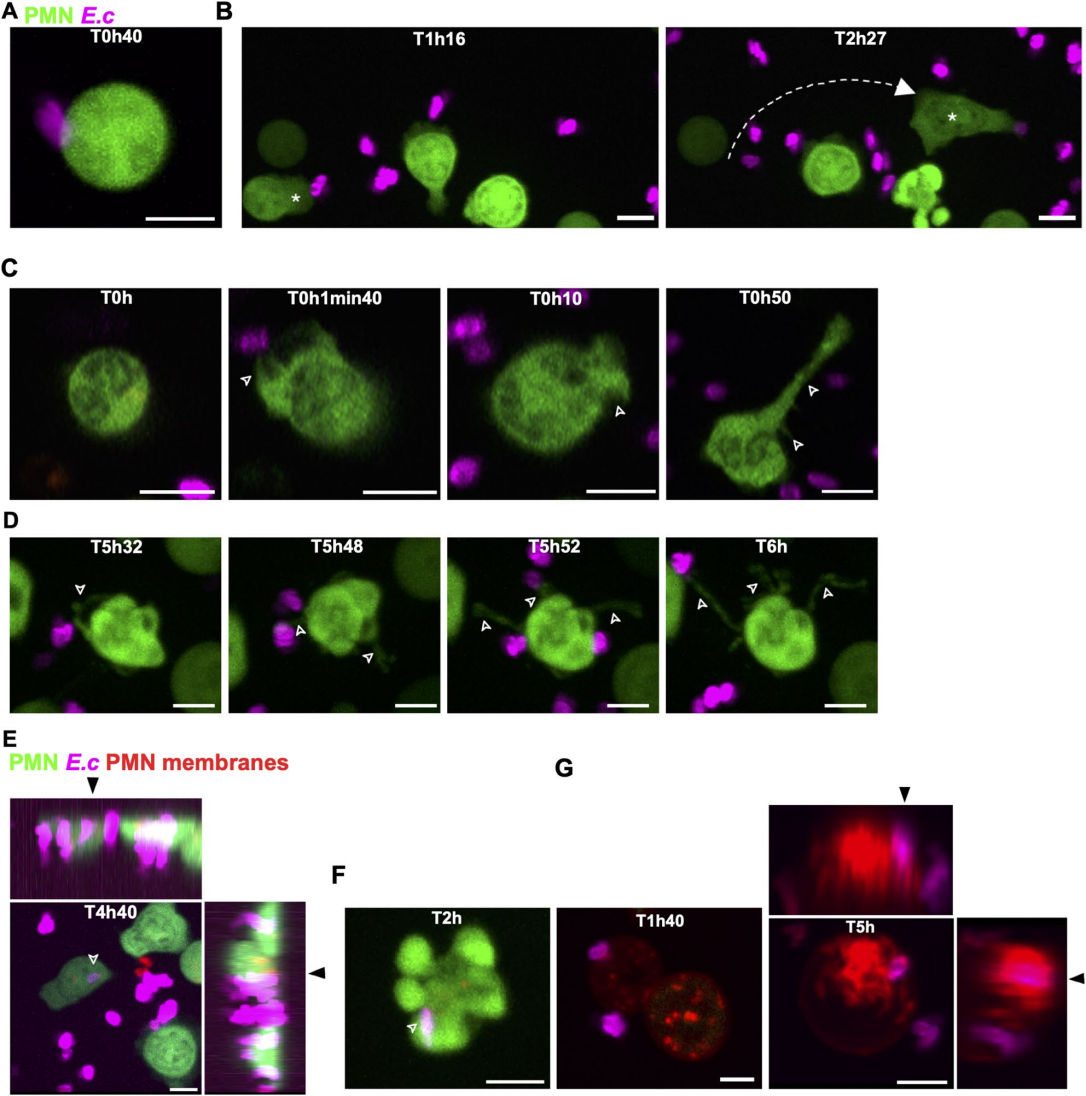
Confocal videomicroscopy images of the dynamics of interactions between PMNs and *E. cuniculi* spores. EGFP^+^ PMNs were incubated with CW^+^ *E. cuniculi* (E.c) spores for 6 h, and their behavior was tracked over time. **A** Round-shaped EGFP^+^ PMN (green fluorescence) interacting with a CW^+^ spore (magenta fluorescence) at an early time point (40 min). **B** Displacement of EGFP^+^ PMN over time (asterisk: starting and arrival point) to make contact with a parasite. **C** and **D** Emission of lamellar pseudopods by EGFP^+^ PMNs over time to interact with and engulf E. cuniculi spores (white arrows). **E**-**G** Maximum projections showing green (EGFP^+^ PMNs), red (PMN membranes), and magenta (CW^+^ *E. cuniculi* spores) fluorescence acquired from videomicroscopy images. **E** An intracellular spore is detected at 4.40h pi (black and white arrows). **F** A dying blebbing PMN containing an intracellular spore at 2h pi (white arrow). **G** Dead PMNs (loss of green fluorescence, persistence of red fluorescence) detected at 1.40h pi and 5h pi (presence of an intracellular spore, black arrows). *n*=4 independent experiments. Scale bar: 5 µm

One of the key functions of PMNs is to engulf and to kill ingested microorganisms at infection sites. Cd11b^+^/Ly6G^+^ double-positive murine PMNs, purified from the bone marrow of LysM-EGFP transgenic mice (Additional file 2: Figure S2), were exposed to Calcofluor White (CW^+^) *E. cuniculi* spores. Their overall behavior and ability to interact with parasites were imaged in real-time over a 6 h period and compared with uninfected PMNs (*n* = 4 independent experiments). Videomicroscopy analysis mainly showed round-shaped cells interacting with spores within the first hour (Fig. 1A). Over time, cells moving toward spores (Fig. 1B) were animated by continuous movements that consisted in change of shape and emission of lamellar pseudopods to interact with and engulf the parasites (Fig. 1C-D, Additional files 3 and 4: Movies S1 and S2 for infected PMNs, and Additional file 5: Movie S5 for uninfected PMNs). The proportion of interacting cells increased with time, leading to successful engulfment events and to the detection of intracellular parasites (Fig. 1E). During the video acquisition time, a proportion of PMNs exhibited signs of cell death, as membrane blebbing (Fig. 1F) and loss of their EGFP fluorescence while retaining the red fluorescent signal (cell membrane labeling with CellMask) (Fig. 1G). Some of these dying or dead PMNs harbored intracellular parasites (Fig. 1F-G).

**Fig. 2:**
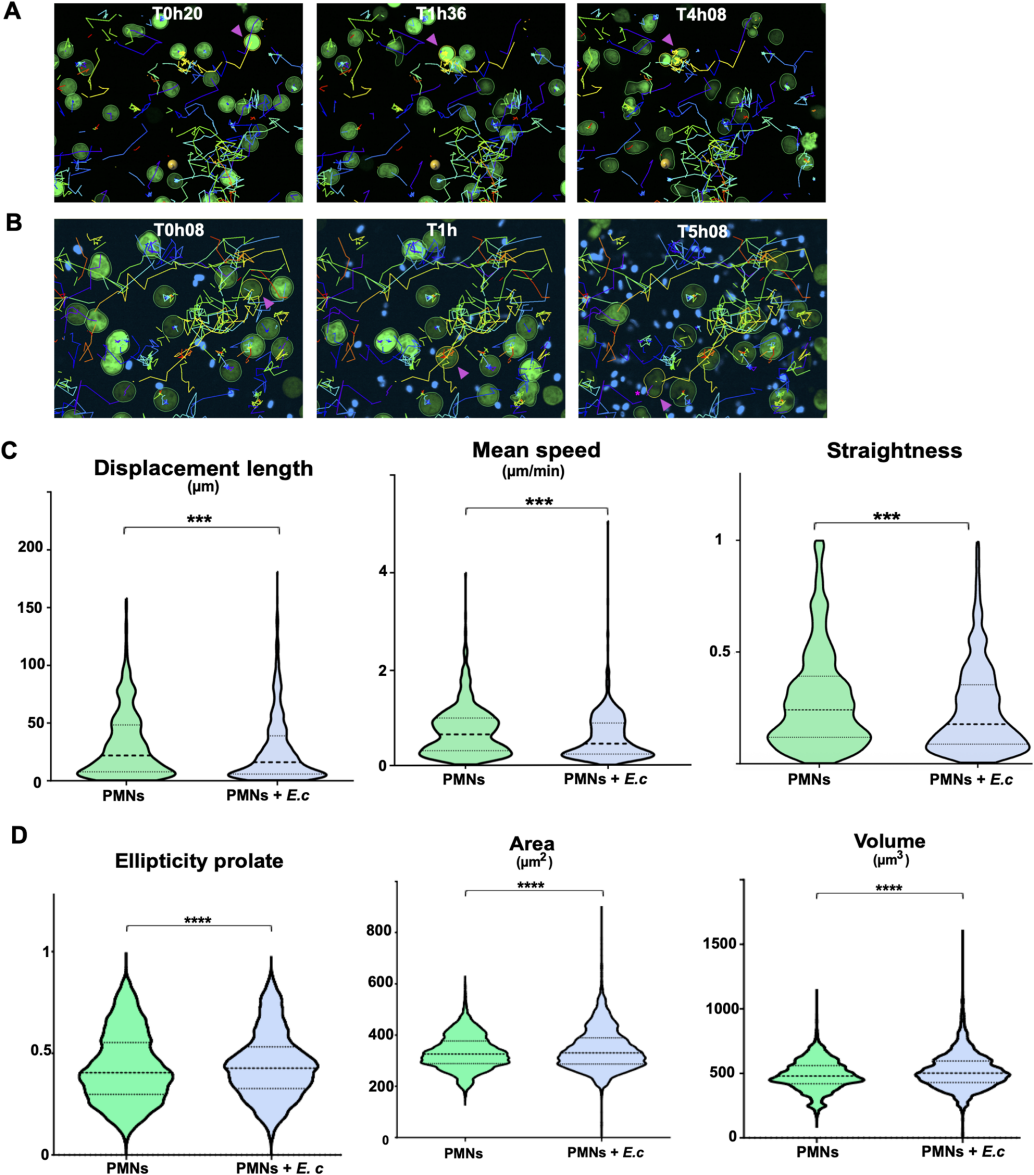
Motility and morphological parameters of PMNs in the presence of *E. cuniculi* spores. EGFP^+^ PMNs were incubated with CW^+^ *E. cuniculi* spores for 6h, and their motility and morphological parameters were measured over time using acquired videos. **A** and **B** Illustrations of EGFP+ PMNs (green) tracking with Imaris software using the “Spots” tool to analyse their motility over time, in absence (**A**) or presence (**B**) of CW+ *E. cuniculi* spores (blue) (0-5h pi) (random fields). Each cell trajectory in the culture medium is represented by a multicoloured line. **A** Tracking of uninfected PMNs displacement. Purple arrows illustrate a cell displacement over time. **B** Tracking of infected PMNs displacement. Purple arrows illustrate a cell displacement over time and its interaction with a CW+ spore at 5h08 pi (pink asterisk). **C** Displacement length, mean speed, and straightness of trajectory of uninfected PMNs (PMNs) or infected PMNs (PMNs + E.c) in contact with parasites. **D** Ellipticity prolate, area, and volume measurements of uninfected PMNs (PMNs) or infected PMNs (PMNs + E.c) in contact with parasites. Data are presented as medians and interquartile ranges pooled from four independent experiments (*n*=4). For motility and morphological parameters, 499 control cells and 843 cells in contact with parasites were analyzed. Statistically significant differences compared to uninfected control PMNs are indicated as follows for all parameters: *p*<0.05 (*), *p*<0.005 (**), *p*<0.001(***) and *p*<0.0001(***). Mann-Whitney one-tailed statistical test

Motility parameters (displacement length, mean speed and linearity of trajectory or straightness) of PMNs in the presence of parasites or cell culture medium (control) were analyzed using Imaris software, based on previously acquired time-lapse videos (Fig. 1). Figure 2A-B illustrates cell trajectories of EGFP+ PMNs in absence (Fig. 2A) or presence (Fig. 2B) of CW + *E. cuniculi* spores, allowing to visualize spore-PMN interactions (Fig. 2B, T5h08, pink asterisk). The motility parameters were quantified between uninfected PMNs and infected PMNs in contact with parasites (Fig. 2C) (*n* = 4 independent experiments, Mann-Whitney one-tailed statistical test). A previously set up analysis protocol was used, and cell trajectories were evaluated over the 6 h acquisition time [10]. Mean speed, displacement length and straightness were significantly decreased for PMNs in contact with parasites (Fig. 2C). These results suggest that cells attracted to parasites experience a slowdown in their movement during the process of catching and engulfing them. Consequently, both the distance traveled (displacement length) and the linearity of trajectory (straightness) were decreased during this interaction.

To further investigate PMNs behavior in the presence of parasites, we calculated a set of metrics characterizing the 3D shape of cells, both in contact with parasites and not, from 1.5 to 6 h post-infection (pi). In this analysis, we used two size indices (volume and surface area) and one shape index (ellipticity prolate). Significant changes of all three morphological parameters were observed following contact (Fig. 2D). The ellipticity prolate index progressively increased over time for PMNs in contact with parasites (significant difference observed at 5–6 h post-contact) (Additional file 6: Figure S3), suggesting that PMNs shapes were stretched in the vertical direction, causing the cells to lose their initial round shape. The mean values for PMNs area and volume were also higher (Fig. 2D), in agreement with the presence of plasma membrane protrusions, as described in Fig. 1A-D. Taken together, these results illustrate that the presence of parasites modifies both the motility and morphological parameters of PMNs.

### PMNs activation in presence of *E. cuniculi* parasites

**Fig. 3:**
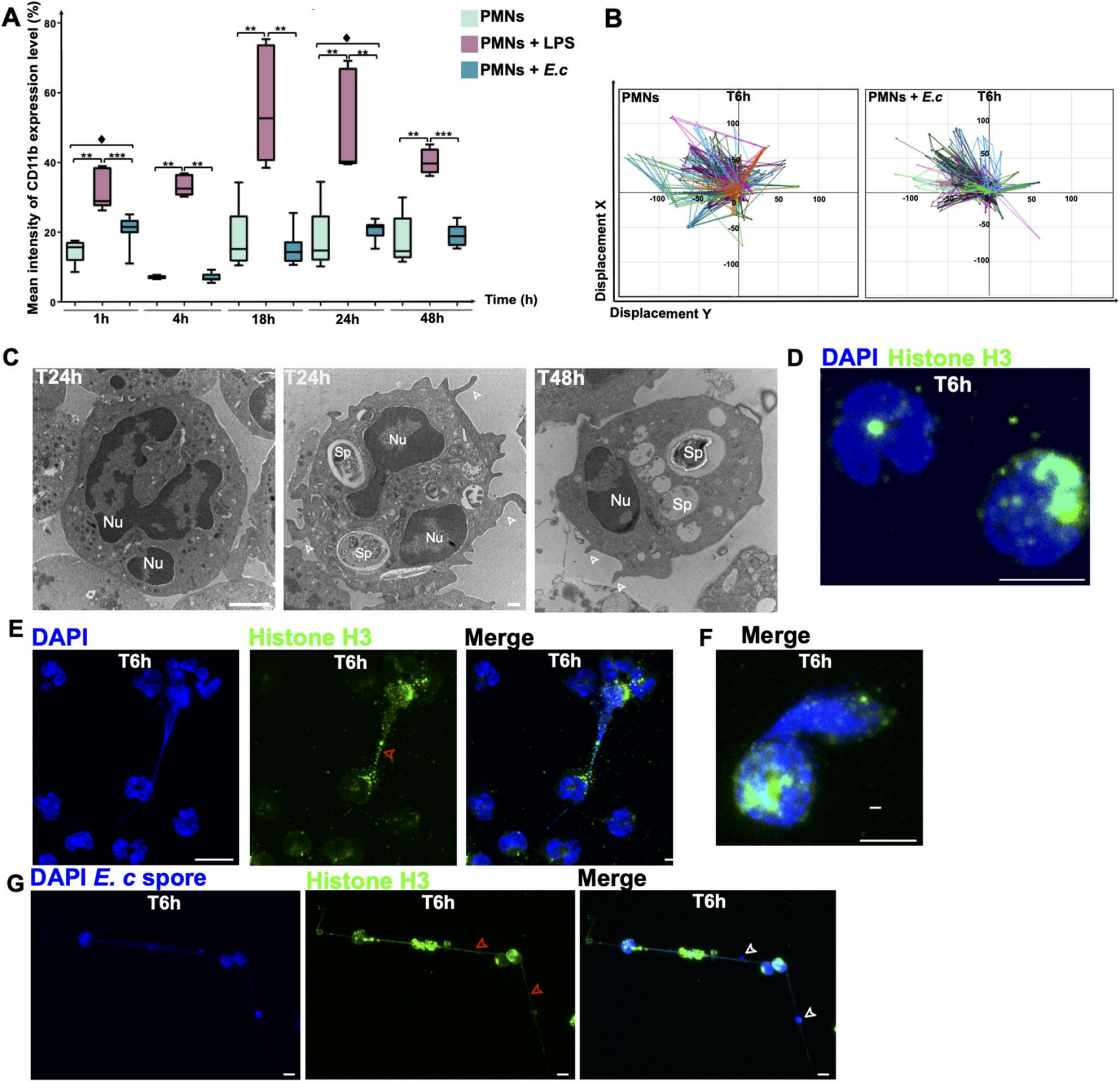
Assessment of PMNs activation in the presence of *E. cuniculi* parasites. Living Ly6G^+^ PMNs were incubated with CW^+^ *E. cuniculi* spores for 48h. **A **CD11b expression levels were measured by flow cytometry in uninfected PMNs (PMNs), LPS-stimulated PMNs (positive control, PMNs + LPS), and *E. cuniculi*-infected PMNs (PMNs + *E.c*) at 1, 4, 18, 24 and 48 h pi. Data are expressed as median and interquartile ranges from 3 (E.c or LPS or PBS) different mice in 3 independent experiments. Statistically significant differences between PMNs, and PMNs + LPS groups: *p*<0.05 (*), *p*<0.005 (**), *p*<0.001(***) and *p*<0.0001(****) or between PMNs, and PMNs + *E.c* groups: *p*<0.05 (⬪). Mann-Whitney one-tailed statistical test. **B** Computed trajectories of PMNs incubated with cell culture medium (control PMNs) or parasites (PMNs + E.c) from 2.5 to 6 h, using videomicroscopy acquisitions. Twenty cells per condition from four independent experiments. Cell positions were normalized to start at the origin (X=0, Y=0). **C** TEM images of uninfected PMNs (left image) or infected PMNs at 24 and 48 h pi (right images). Degraded spores are detected inside phagosomes of infected PMNs. Infected cells emit pseudopods (white arrowheads). Scale bars: 2 µm (uninfected PMNs) and 500 nm (infected PMNs). *n*=2. Nu = nucleus; Sp = spore. **D**-**E** Confocal immunofluorescence images of infected PMNs at 6h pi to visualize the formation of NETs after anti-citrunillated Histone H3 (**D**) and anti-elastase immunolabelings (**E**). Blue fluorescence signal: cell nuclei (DAPI) and spores (CW^+^), green fluorescent signal: PMNs citrunillated histone H3, yellow signal: elastase. In the presence of parasites, PMNs expel NETs (red arrows) (**D** and **E**), which sometimes come into contact with spores (E, white arrows). Scale bar = 5 μm, *n*= 4 independent experiments

PMNs are appropriately armed to help clear mammalian-invading parasites through numerous microbicidal mechanisms [14]. Additional experiments were carried out to examine the functional properties of purified Ly6G^+^ PMNs in the presence of *E. cuniculi* spores. In the first set of experiments, their activation was assessed at 1, 4, 18, 24, and 48 h after contact, based on the expression of the activation marker integrin receptor CD11b (Additional file 2: Figure S2A). A slight increase of CD11b expression level was observed at the very early time point (1 h pi) and at 24 h pi, compared to uninfected PMNs (Fig. 3A) (*n* = 3 independent experiments, Mann-Whitney one-tailed statistical test). In parallel, cytokines were quantitated in the supernatants of infected or uninfected PMNs. No characteristic pro-inflammatory (TNFα, IFNg, IL-2, IL-5, IL-6, IL-12) or anti-inflammatory (IL-4, IL-10) cytokine profile was detected over time (Additional file 7: Figure S4). Nevertheless, we can notice that the presence of parasites is not responsible for a strong inflammatory response at early time-points (TNFα, IFNγ, IL-6), with only a trend to increase for TNFα at 18 h pi (*p* = 0,07) (Additional file 7: Figure S4). Figure 3B illustrates the computed trajectories of PMNs incubated with either parasites or cell culture medium (control) for 6 h. As described in Fig. 2C, PMNs in the presence of parasites traveled shorter distances, and remained closer to their starting points, indicating a behavioral modification to promote interaction with the parasites (Fig. 3B) (*n* = 4 independent experiments).

In another set of experiments, the microbicidal properties of infected PMNs were assessed. Their phagocytic activity was analyzed at 24 and 48 h pi by TEM, revealing degraded spores (crenellated wall, loss of spore internal content) inside large phagolysosomes (Fig. 3C, Additional file 8: Figure S5A). Some dying PMNs harboring intracellular spores inside phagosomes presented abnormal nucleus morphology: small, fragmented and eccentric nuclei with condensed chromatin (Fig. 3C, 48 h pi, and Additional file 8: Figure S5B). At 24 h pi, TEM quantification of spore degradation was estimated for PMNs that harbored morphological detectable spores inside phagosomes, based on the two morphological criteria described above (*n* = 2 independent experiments, 378 infected cells analyzed). The analysis revealed a total proportion (%) of spores that lost their internal content, associated or not to a visible crenellated wall (Additional file 8: Figure S5A, 24 h pi), equal to 78,79 ± 9,94%. In other phagolysosomes, the parasite was fully degraded (Fig. 3C, 48 h pi and Additional file 8: Figure S5A), illustrating that the engulfment process is mostly successful and leads to parasite killing.

The ability of *E. cuniculi* infected PMNs to trigger NETs formation, another key effector mechanism used by PMNs to kill pathogens extracellularly, was further assessed. In presence of PMA, a strong inducer of NETs formation, NETs were identified after anti-Histone H3 specific immunolabelling (positive control) (Additional file 8: Figure S5D, red arrow), compared to uninfected PMNs (Additional file 8: Figure S5C, negative control). Interestingly, the presence of spores also triggered the formation of extracellular fibers at 6 h pi, further identified as NETs after anti-Histone H3, anti-citrullinated Histone H3 and anti-elastase specific immunolabeling (Fig. 3D and E and Additional file 8: Figure S5E, red arrows). Figure 3E (image on the right) and supplemental Figure S5E highlight the interaction between NETs and CW^+^ *E. cuniculi* spores (white arrows). Quantitative data revealed a trend to increase (*p* = 0.06) for NETs production by PMNs in presence of parasites, compared to uninfected PMNs (Additional file 8: Figure S5F) (*n* = 4 independent experiments, binomial GLMM statistical test in R).

### Diversity of interactions between *E. cuniculi* parasites and PMNs

**Fig. 4:**
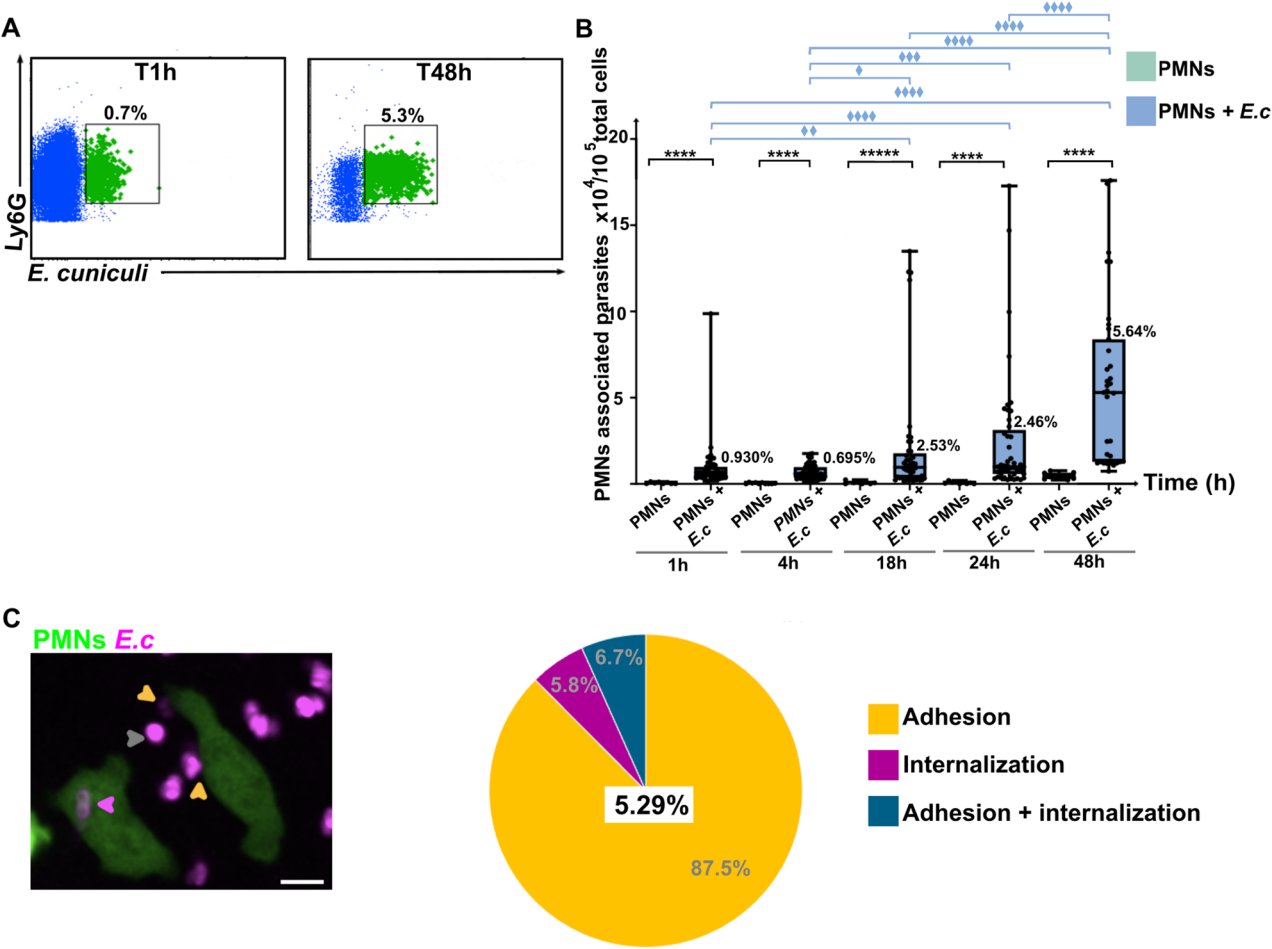
In vitro interactions between PMNs and *E. cuniculi* parasites. Living CD11b^+^Ly6G^+^PMNs were incubated with CW^+^*E. cuniculi* spores for 48 h.** A**-**B** Flow cytometric analysis showing the proportion of cells interacting with parasites at 1, 4, 18 and 48 h pi. **A** Representative dot plots and percentages of parasite-associated cells (rectangles) are shown from infected PMN at 1 h and 48 h pi. **B** Total number of PMNs among live cells interacting with parasites from 1 h to 48 h pi (PMNs + *E.c*), compared to uninfected PMNs (PMNs) (*) or between infected PMNs (⬪). Data are expressed as median and interquartile ranges from 8 independent experiments. 4-12 (*E.c*) or 1-2 (control) samples per experiment. Mean values were reported on the infected group graph. Statistically significant differences: *p*<0.0001(****), *p*<0,05 (⬪),*p*<0,01 (⬪⬪), *p*<0,001 (⬪⬪⬪), *p*<0.0001(⬪⬪⬪⬪ or ****), Mann-Whitney one-tailed statistical test. **C** Detection and quantification of interaction events from acquired videos using Imaris software. Total interaction events between EGFP^+^PMNs (green fluorescence signal) and CW^+^*E.c* parasites (magenta fluorescence signal) (5.29%, corresponding to 1.68.104 spores out of 3.92.105 total spores), adhesion events (87.25%; orange arrows), invasion events (5.8%; pink arrows), and simultaneous adhesion/invasion events (6.7%). Non-interacting cells are indicated by grey arrows. A total of 843 cells were in contact with parasites during the 6 h acquisition period. *n*=4 independent experiments. Scale bar= 5 µm

**Fig. 5:**
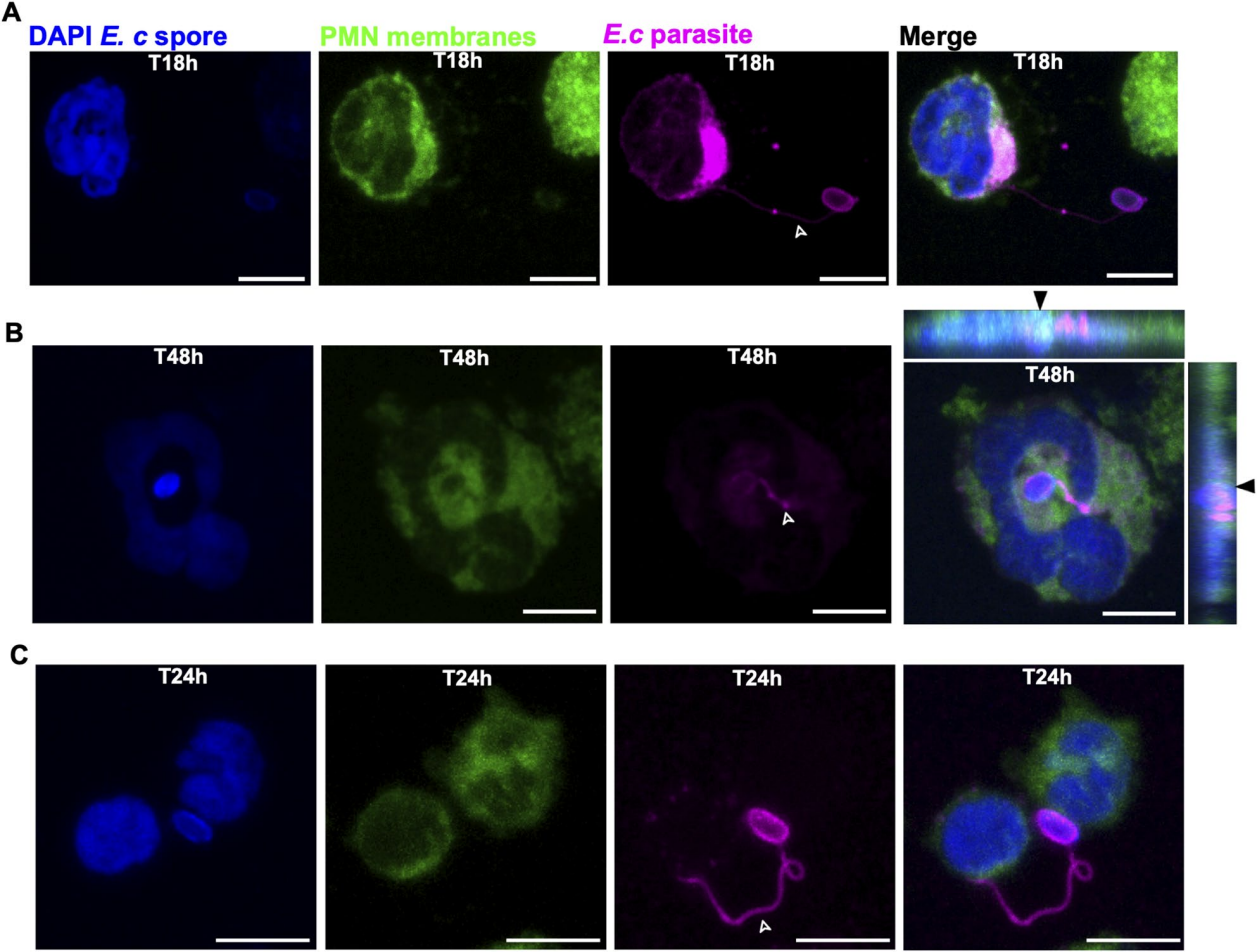
*E. cuniculi* spores extrude their polar tube to interact with PMNs. Living CD11b^+^Ly6G^+^ PMNs were incubated with CW^+^ *E. cuniculi* spores for 48 h. Confocal immunofluorescence images showing spores extruding their polar tube to infect PMNs at 18, 24 and 48 h pi. Blue fluorescence signal: cell nuclei (DAPI) and spores (CW^+^), green fluorescence signal: PMN membranes, magenta fluorescence signal: *E. cuniculi* parasites (all parasitic stages). **A **An extracellular spore extrudes its polar tube (white arrow) to interact with a PMN. **B** An intracellular spore (black arrows) extrudes its polar tube (white arrow) inside a PMN. **C **An adherent spore extrudes its polar tube (white arrow) to infect a neighboring cell. Scale bar = 5 μm (*n* = 3)

To further analyze the interactions between PMNs and parasites, purified murine PMNs were exposed to CW^+^ *E. cuniculi* spores (Multiplicity of infection, MOI 5), and the proportion of parasites associated with live Ly6G^+^ PMNs was measured at 1, 4, 18, 24, and 48 h pi under static conditions using FACS analysis (Fig. 4A-B and Additional file 9: Figure S6) (*n* = 8 independent experiments, Mann-Whitney one-tailed statistical test). Surprisingly, despite PMNs being professional phagocytes, only a small proportion of association events were detected at early time points (0.930 ± 1.44 and 0.695 ± 0.422 at 1 and 4 h pi, respectively). From 1 to 48 h pi, this proportion increased significantly (Fig. 4A-B and Additional file 9: Figure S6B) and correlated with a marked increase in cell mortality over time (Additional file 9: Figure S6C). When comparing the different time-points inside the *E. cuniculi* PMNs infected group, significant differences were observed between the early (1 and 4 h) and late time-points (18, 24, 48 h). In parallel, further analyses were performed on the acquired videos presented in Figs. 1 and 2, to analyze the nature of the early spore-PMN interactions during the 6-hour acquisition period by videomicroscopy (MOI 10). Contact interactions were observed, including adhesion (yellow arrowhead), internalization (pink arrowhead), and adhesion/internalization events (Fig. 4C). Quantitative data were obtained by counting the number of parasites interacting with cells during the 6-hour acquisition period (distinguishing between intracellular and adherent parasites) and comparing them to the total number of parasites identified in each field. Each parasite was counted independently in each video frame. The proportion of total interaction events remained low (5.29%), with the majority being adhesion events (87.5%), followed by internalization (5.8%) and combined adhesion/internalization events (6.7%) (Fig. 4C, pie chart representation). In addition, a high proportion of cells interacted with parasites over the 6-hours acquisition period, with a total number of cells harbouring intracellular parasites or both intracellular and adherent spores, compared to total number of cells analyzed, equal to 45,66 ± 2,17% (*n* = 4 independent experiments; 990 cells analyzed in total). Microsporidia are known for their unique ability to rapidly inject their infectious spore content into the target cell via a unique and highly specialized organelle, the polar tube (PT). Therefore, direct contact between the spore and the cell is not required in some interactions [22]. Figure 5 provides the first visual evidence of spores extruding their PT for contact with a PMN (Fig. 5A, arrow) or inside a PMN (Fig. 5B, arrow and Additional file 10: Figure S7A). In some cases, spores adhered to a PMN and extruded their PT for contact with a neighboring cell (Fig. 5C), therefore increasing the probability of a successful internalization event [23] (*n* = 3 independent experiments).

### *E. cuniculi* parasites can initiate a development inside PMNs in vitro

**Fig. 6:**
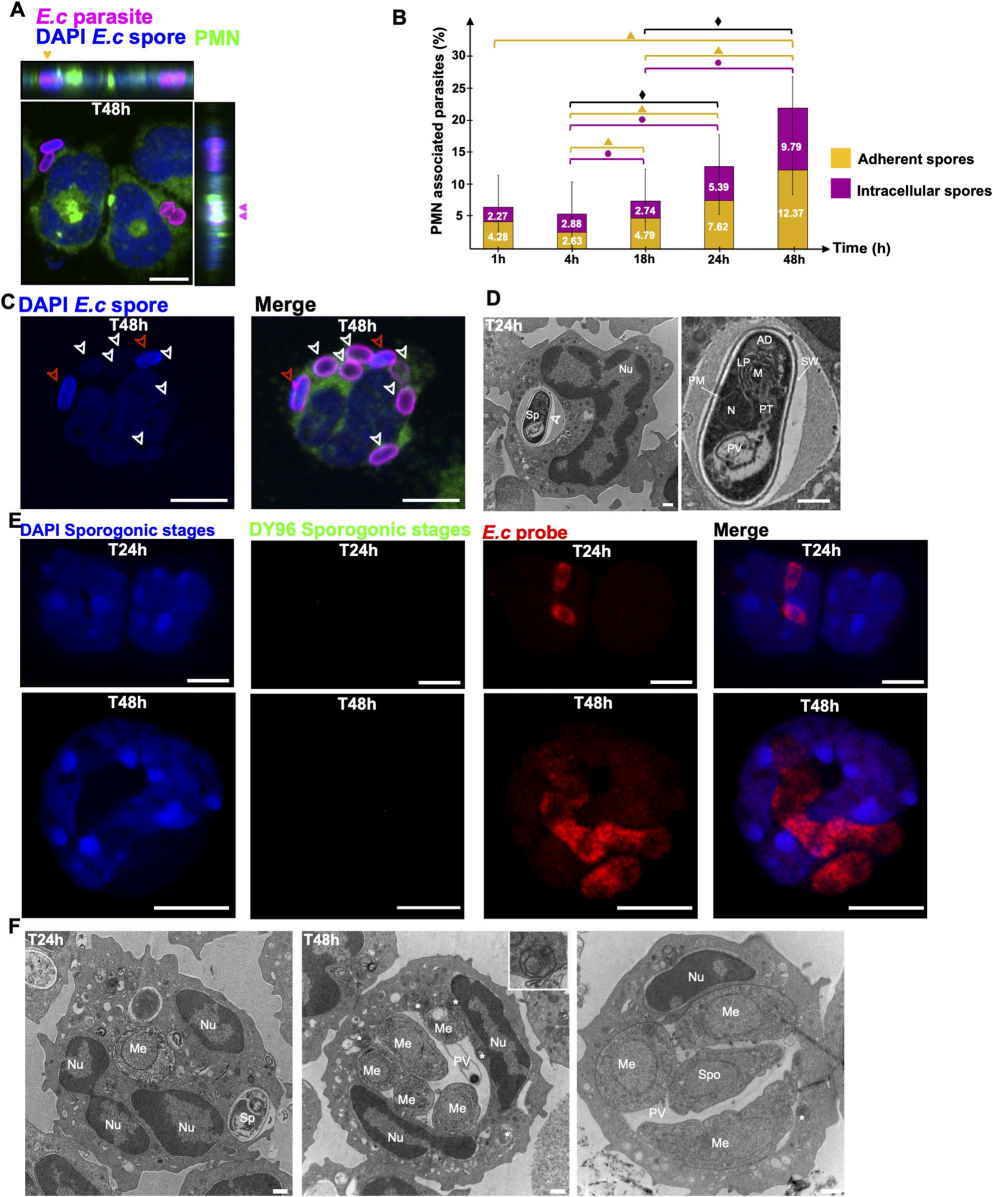
*E. cuniculi *parasites initiate development inside PMNs in vitro. Living CD11b^+^Ly6G^+^ PMNs were incubated with CW^+^ *E. cuniculi* spores (**A**-**B**) Immunostainings performed on infected PMNs at 1, 4, 18, 24 and 48 h pi. Fluorescence signals are blue: cell nuclei (DAPI) and spores (CW^+^), green: PMN membranes (Cytopainter) or magenta: *E. cuniculi* parasites (all parasitic stages). **A** Confocal immunofluorescence image showing adherent (yellow arrow) and intracellular (purple arrow) spores. **B** Quantitative analysis of the proportion of adherent and intracellular parasites over time. Data are expressed as median and interquartile ranges from 4 independent experiments. Statistically significant differences (*p*<0.05) indicated as the proportion of: (▲) adherent spores, (●) intracellular spores, (◆) global interactions (adhesion + internalization), in the infected group over time. **C** Confocal microscopy image of degraded spores (white arrows, loss of CW^+^ signal) and undegraded spores (red arrows, intact CW^+^ signal) inside a PMN. **D** TEM image of an infected PMN harboring an intact spore inside a phagosome 24 h pi (insert). The coiled polar tube is visible inside the spore (white arrow). Intact spore structure (insert): spore wall (SW), plasma membrane (PM), posterior vacuole (PV), nucleus (N), anchoring disc (AD), polar tube (PT), manubroid (M) and lamellar part of the polaroplast (LP). **E** Confocal microscopy images of infected PMNs (24 and 48 h pi) after FISH labeling. Blue signal: cell nuclei (DAPI) and spores (CW^+^), green signal (DY96): counterstaining, red signal: developing parasites (*E. cuniculi* probe). **A**, **C**, **D**, **E** Scale bar = 5 μm. **F** TEM images showing spores (Sp) and developing parasites inside phagosomes of infected PMN (24 and 48 h pi). Early stages of *E. cuniculi* development: meronts (Me) (24 and 48 h pi), and a sporont (Spo) (48 h pi) inside phagosomes. Several mitochondria (asterisks, inset) are adjacent to the PV membrane. Nu: nucleus. Scale bar = 500 nm

To further analyze the fate of parasites after contact with PMNs, purified cells were exposed to CW^+^ *E. cuniculi* spores under static conditions at MOI 5. Specific immunolabeling of PMNs membranes and *E. cuniculi* parasites was performed to quantify the proportion of adherent (orange arrowhead) and intracellular (pink arrowheads) parasites (Fig. 6A) at 1, 4, 18, 24, and 48 h pi, and to analyze the different fates of intracellular parasites (*n* = 4 independent experiments, Mann-Whitney one-tailed statistical test). Comparable experiments were performed on Human Foreskin Fibroblast (HFF) cells at the same time points and MOI 5 (*n* = 3 independent experiments, Mann-Whitney one-tailed statistical test). The HFF cell line served as the reference, as microsporidia complete their life cycle inside these cells (Additional file 11: Figure S8A). Surprisingly, the global proportion of spores interacting with PMNs (Fig. 6B) was lower than with HFF cells at early time points (1 and 4 h pi) (Additional file 11: Figure S8B). PMN adhesion events remained comparable until 4 h pi, then increased from 18 to 48 h pi (with a significant increase at 18 and 48 h pi). The proportion of internalization events remained stable until 18 h pi, then increased after 24 h pi, with a significant rise at 48 h pi (Fig. 6A-B). Interestingly, some intact spores were detected inside PMN at 24 and 48 h pi (Fig. 6C red arrows, Fig. 6D: intact spore wall and coiled region of the PT visible, insert and Additional file 10: Figure S7B, white arrowhead), illustrating that some parasites could escape degradation inside PMNs. Given the short half-life of PMNs, new infections could have occurred at later time points following the lysis of dying PMNs and the release of intracellular infectious spores into the culture medium. Unlike HFF cells (Additional file 11: Figure S8C), no infectious foci were detected inside PMNs between 18 and 48 h pi. However, using fluorescence in situ hybridization (FISH), developing parasites were occasionally observed at 24 and 48 h pi. This is the first time that *E. cuniculi* parasites have been shown to initiate development inside PMNs (Fig. 6E and Additional file 10: Figure S7C). At 48 h pi, the proportion of these developing parasites inside PMNs was equal to 1,4 ± 0,4% (*n* = 4). Ultrastructural analysis revealed early stages of parasite development inside phagosomes, including meronts and sporonts (Fig. 6F), with mitochondria closely associated with the phagosome membrane (Fig. 6F, asterisks, insert).

### *E. cuniculi* parasites can initiate a development inside PMNs in vivo

**Fig. 7:**
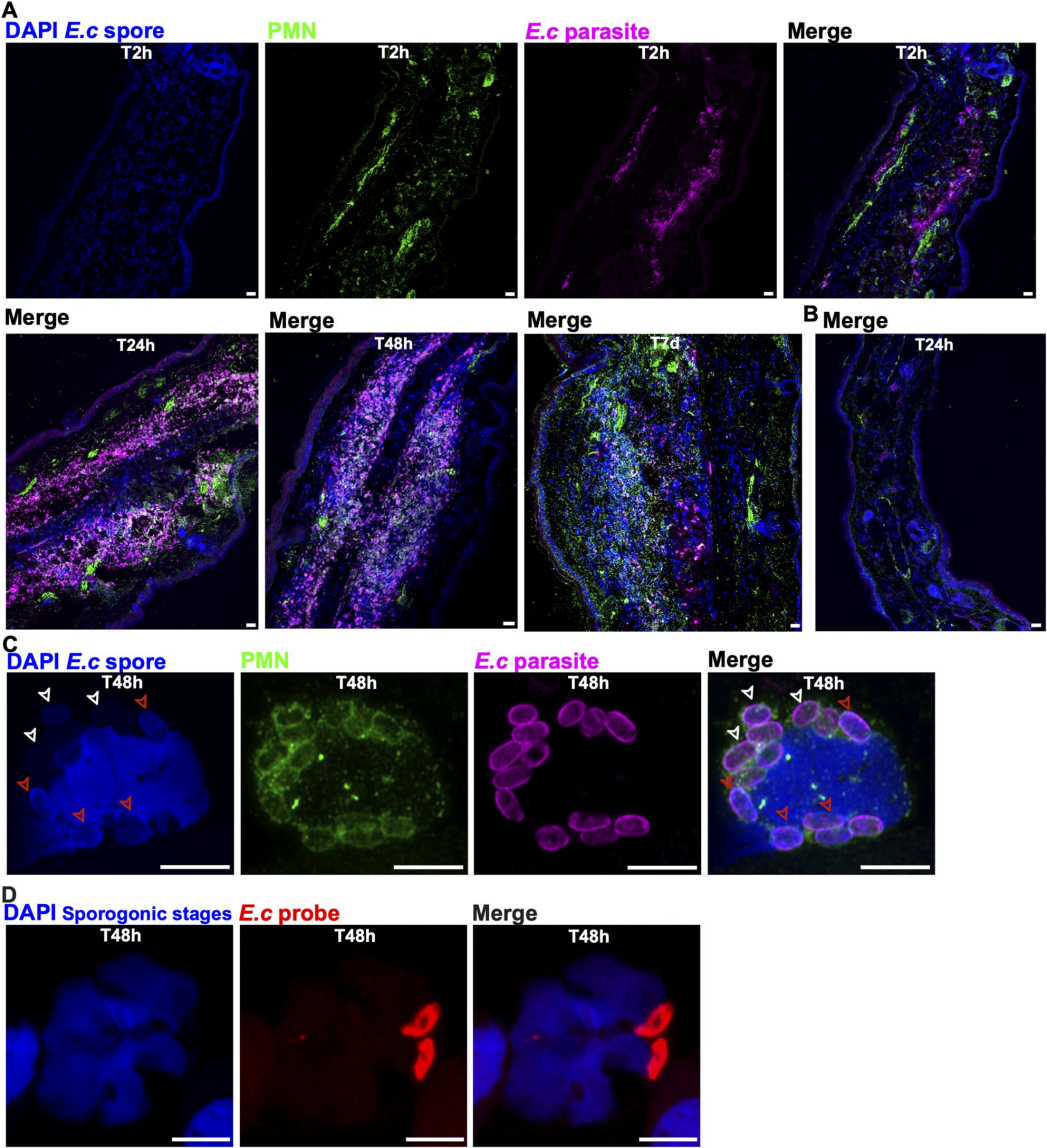
Encephalitozoon cuniculi parasite development inside PMNs following micro-injection of spores into the mouse ear pinna. Confocal immunofluorescence images of immunolabeled cryosections of infected ear tissues. LysM-EGFP mice (EGFP^+^ phagocytes) were inoculated with fluorescent *E. cuniculi* spores (CW^+^, blue fluorescence signal). Immunostaining was performed on cryosections collected from 2 h to 7 days (7d) pi (**A**-**B**). Green, pink, and blue fluorescence signals correspond to specific labeling of inflammatory cells as PMNs (NIMPR14, sc-59338, mAb), all parasitic stages (anti-*E. cuniculi* polyclonal serum), and cell nuclei (DAPI)/spores (CW), respectively. **A** *E. cuniculi*-infected mice. **B** Control mice inoculated with PBS in the ear pinna tissue. Scale bar = 10 μm. **C** Confocal microscopy images of immunostainings performed on *E. cunicul*i-infected PMNs purified from the ear tissue of LysM-EGFP infected mice at 48 h pi. Blue, green, and magenta fluorescence signals correspond to cell nuclei (DAPI) and spores (CW^+^), PMNs, and all parasitic stages (anti-*E. cuniculi* polyclonal serum), respectively. Intact spores are detected inside purified PMNs (red arrows), as well as degraded spores (loss of the CW^+^ signal, white arrows). **D** Confocal microscopy image of an infected PMN purified from the ear tissue of infected mice at 48 h pi, after FISH labeling. Blue fluorescence signal: cell nuclei (DAPI), red fluorescence signal: developing parasites (*E. cuniculi* probe). Developing intracellular parasites are detected inside PMNs. Scale bar = 5 μm, *n*=3 independent experiments

In vivo, *E. cuniculi* parasites induced an important inflammatory recruitment of phagocytes in the mouse ear pinna model, with intracellular parasites detected inside the recruited phagocytes up to 72 h pi [10]. In a new set of experiments, LysM-EGFP mice were intradermally inoculated in the ear pinna with 10^7^ *E. cuniculi* spores or PBS (control mice). Immunolabeling was performed on cryosections of infected tissues from 2 h to 7 days pi to track inflammatory cells recruitment over time (*n* = 3 independent experiments). Phagocytes were continuously recruited to the injection site (Fig. 7A and Additional file 12: Figure S9A), compared to the control mice (Fig. 7B and Additional file 12: Figure S9B at time-points 24 h and 7 days). This result was consistent with the ability of parasites to persist and develop within the mouse cutaneous tissue [10]. To assess whether parasites could specifically persist inside PMNs in vivo, Ly6G^+^ cells were purified from the infected cutaneous tissues and subsequently stained using *E. cuniculi*-specific polyclonal antibodies and a cell membrane marker. Intact spores were detected intracellularly at 48 h pi (Fig. 7C and Additional file 13: Figure S10A). Additionally, using FISH, some developing parasites were detected at the same time point, supporting our in vitro data (Fig. 7D and Additional file 13: Figure S10B). These results suggest that PMNs can act as carriers for viable (whether developing or not) parasites.

### Dying PMNs silently transfer *E. cuniculi* parasites to macrophages or fibroblast cells

**Fig. 8:**
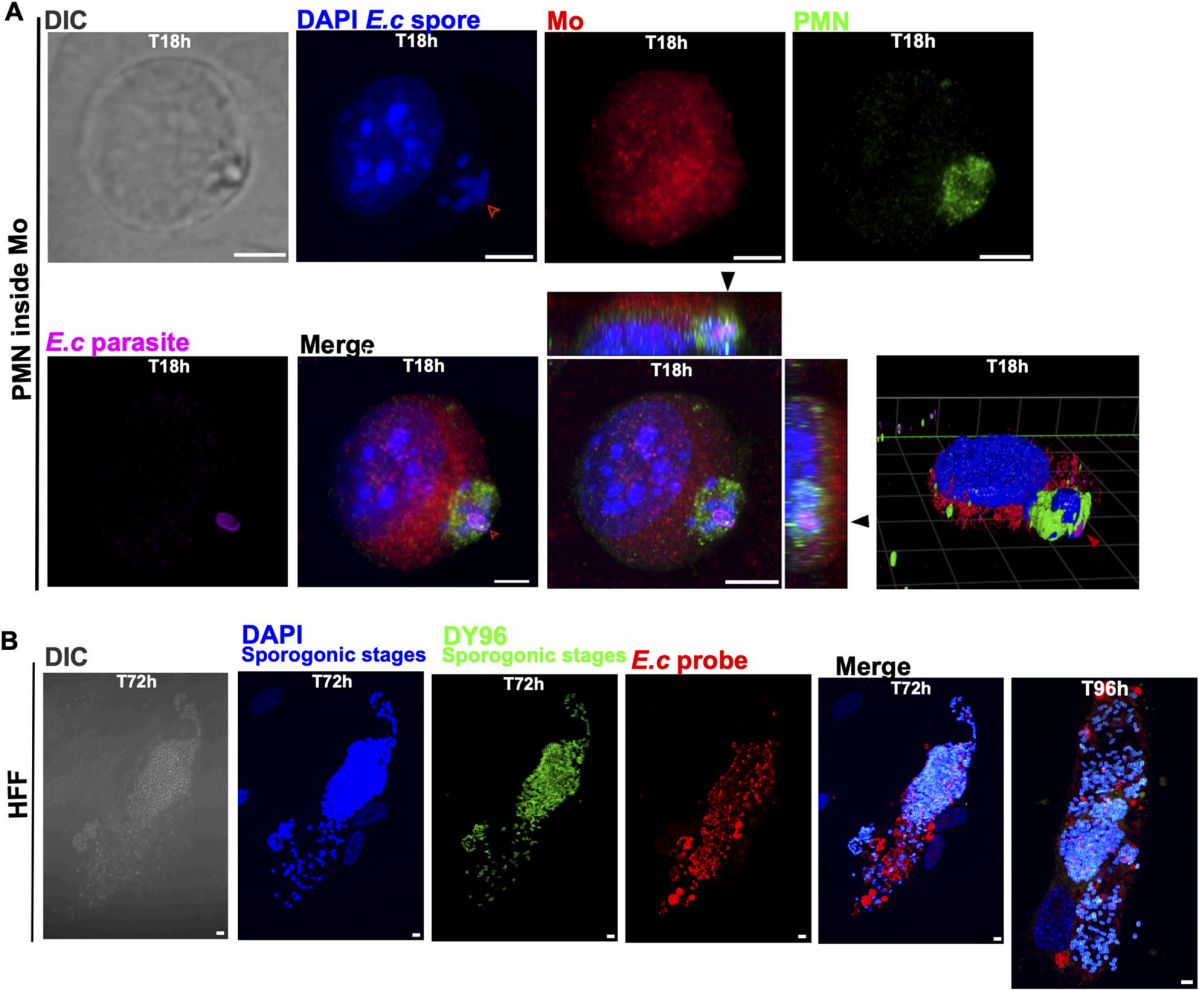
Phagocytosis of dying PMNs harboring intracellular *E. cuniculi* by MOs and HFF. **A **Confocal immunofluorescence image of immunolabelings performed on J774 murine MOs co-incubated with *E. cuniculi*-loaded PMNs for 18 h. DIC: transmitted light. Blue, red, green, and magenta fluorescence signals correspond to cell nuclei (DAPI), spores (CW^+^), J774 murine MO F4/80-specific marker, NIMPR14 mAb used as a marker of inflammatory cells as PMNs, and all parasitic stages (anti-*E. cuniculi* polyclonal serum), respectively. One representative image of a dying PMN containing an intact spore being internalized by a MO. Scale bar = 5 μm, *n*=3 independent experiments. **B** FISH labelling. DIC: transmitted light, blue fluorescence signal: cell nuclei (DAPI) and sporogonic stages, green fluorescence signal (DY96): counterstaining, red fluorescence signal: developing parasites (*E. cuniculi* probe). Confocal immunofluorescence image of HFF cells co-incubated during 18h with *E. cuniculi*-loaded PMNs. Scale bar = 5 μm. *n*=3 independent experiments

PMNs are short-lived phagocytic leukocytes, and in mammals, dying PMNs are mainly cleared by MOs [24, 25]. While some *E. cuniculi* parasites were highly degraded inside PMNs, others remained intact at 24 and 48 h pi. When incubated with murine MOs, parasite-loaded PMNs were engulfed by the MOs. In the representative example shown in Fig. 8A, a CW^+^ *E. cuniculi* spore maintained its intact morphology inside a dying PMN which exhibited nuclear fragmentation (Fig. 8A and Additional file 14: Figure S11A) (*n* = 3 independent experiments). Complementary experiments showed that *E. cuniculi* parasites can achieve a complete development inside J774 MOs, with DY96 + neoformed spores detected at 72 h pi (Additional file 14: Figure S11B and S11C) (*n* = 3 independent experiments). Thus, MOs are appropriate shuttles for further intracellular development of parasites entered via dying PMNs phagocytosis. In another set of experiments, HFF cells were co-incubated with sorted *E. cuniculi*–loaded PMNs (Fig. 8B and Additional file 15: Figure S12A) (*n* = 3 independent experiments). Presence (Additional file 15: Figure S12B) or absence (Additional file 15: Figure S12C) of extracellular spores was assessed by TEM analysis in the PMNs samples respectively before and after sorting. Parasites completed their infectious life cycle inside HFF cells after co-incubation with sorted *E. cuniculi*–loaded PMNs (Fig. 8C and Additional file 15: Figure S12A). Infectious foci numbers inside HFF cells were respectively equal to 1,8 ± 1,1% and 1,1 ± 0,7% at 72 and 96 h post-incubation with infected PMNs (*n* = 3). This result illustrates that spores remain infectious inside infected PMNs.

## Discussion

PMNs represent the most abundant immune cells in human blood and serve as key effector cells of the innate immune system, mostly recognized for their highly effective antimicrobial properties. They are rapidly mobilized to infectious sites to attack and eliminate invading pathogens through mechanisms such as phagocytosis, degranulation, and NETs formation. This heterogeneous population of leukocytes can also regulate adaptive immunity, particularly by affecting T cell and DCs functionality [14]. However, little data concerning the role of PMNs during microsporidiosis are available. Histological studies of human infections reveal inflammatory infiltrates predominantly composed of phagocytes (mononuclear cells and PMNs), some of which contain microsporidian spores [26]. In other vertebrate models, such as fish and mice, PMNs are quickly recruited to infection sites together with MOs and actively participate in spore elimination. Despite their strong microbicidal properties, PMNs sometimes fail to completely destroy spores, potentially contributing to parasite persistence within the infected host [1, 27]. To improve our understanding of the role of PMNs during microsporidiosis, we investigated the dynamics of PMN/parasite interactions both in vitro and in vivo using the murine ear pinna model previously developed in our laboratory, combining live and static cell imaging approaches. In particular, we examined not only the effects of PMNs on *E. cuniculi* parasites, but also the effects of parasite on PMNs, including host cell invasion, potential use of PMNs as an intracellular niche for parasite development, and/or their involvement in the silent transfer of spores to other host cell types such as MOs or fibroblasts).

In this study, mature murine PMNs were purified from the bone marrow, their main site of production, where they are produced at a rate of approximately 10^7^ cells per day and undergo maturation over 2–3 days. Working with this cell type represents a technical challenge [14], as PMNs are short-lived cells. The literature on their half-life is still controversial, with estimates ranging from 10 to 13 h in the circulation to 8–18 h in tissues [28]. However, priming or activation of PMN in response to various mediators during acute or chronic inflammatory conditions can extend their lifespan [29]. Consistent with these observations, PMN/Microsporidia interactions, which occur in an inflammatory context associated with PMN activation, were analyzed up to 48 h pi, but not beyond, as 72 h pi was not compatible with sustained PMN viability. In our experiments, we obtained a high purity level, with 97% viable CD11b^+^ cells collected from the bone marrow, including 91% CD11b^+^Ly6G^+^ PMNs displaying the typical multilobed nucleus. This ratio is consistent with that reported in a previous study, which obtained 92.1% Cd11b^+^Ly6G^+^ purified cells) [30].

The effects of *E. cuniculi* parasites on PMNs were first investigated. Exposure of PMNs to *E. cuniculi* spores altered their overall behavior in vitro. Over time, some cells lost their initial round shape and deformed to move towards and capture the parasites, as expected [31]. In presence of parasites, PMNs predominantly emitted lamellar pseudopods, a characteristic response observed in vitro [32–35]. These thin and flat protrusions facilitated their adhesion to the cell culture surface and their migration towards spores to interact with them [36, 37]. Additionally, long and slender cellular protrusions were observed, suggesting the presence of filipodia (Fig. 1D, images at 5h52 and 6 h pi) [34]. These protrusions help migratory cells to sense and explore their environment. As experiments were performed in vitro, and according to the literature, filipodia were likely present in only a small proportion of cells [32]. Quantitative analysis of PMNs movement parameters (mean speed, displacement length, and trajectory straightness) confirmed that, in the presence of spores, cells modified their behavior to enhance interactions. When interacting with spores, PMNs indeed slowed down, decreased their displacement length, and followed less linear trajectories. Similar results were observed in vivo in the ear pinna model, where EGFP^+^ phagocytes showed reduced average speed after inoculating mice with *Staphylococcus aureus* biofilms or *E. cuniculi* spores in the cutaneous tissue [10, 21]. The “quick” cell phenotype (rapid EGFP+ phagocytes with linear trajectories) identified in the Microsporidia infection model was not found in vitro, highlighting a limitation of this experimental approach, where chemotactic signals typically produced in vivo by immune cells to promote cell movement towards other target tissues are missing [10]. In the bloodstream, PMNs are classically round-shaped [38]. Quantitative analysis of infected PMNs morphological properties (ellipticity prolate, area, volume) confirmed that in the presence of spores, PMNs deformed by emitting pseudopods, leading to an increase in both their volume and area. This phenomenon was also reported in a mouse femur model of *S. aureus*-infected bone implants [39]. Taken together, these results are consistent with the classical PMNs transition from a spherical to a flattened morphology, marking the initial step before they migrate from the bloodstream into extravascular tissues [38]. After contact, lamellipodia facilitated spore capture, leading to the formation of pseudopodia and internalization of parasites within phagosomes, corresponding to the second key morphological change undergone by PMNs [38].

In the presence of *E. cuniculi* parasites, PMNs activated two main microbicidal mechanisms: phagocytosis and the release of NETs [40]. After engulfment, parasite elimination was usually effective, with numerous spores losing their ovoid shape and exhibiting altered walls and degraded internal contents within phagolysosomes, as previously described [41]. The presence of *E. cuniculi* spores also triggered the formation of extracellular web-like structures composed of decondensed chromatin decorated with antimicrobial proteins, including PMN elastase and citrullinated histones. These observations provide the first direct evidence of NETosis during microsporidian infection. According to the literature, NET formation can proceed via multiple mechanisms depending on the activating stimulus and the signaling pathways engaged. The classical ROS-dependent pathway, known as suicidal NETosis, culminates in PMNs lysis and the release of nuclear chromatin. By contrast, vital NETosis allows PMNs to rapidly release NETs while preserving plasma membrane integrity and maintaining essential cellular functions. Moreover, PMNs can produce mitochondrial NETs, in which mitochondrial DNA rather than nuclear chromatin forms the extracellular scaffold [42–45]. NETosis has previously been described in response to several parasites, including *Leishmania major* and *Toxoplasma gondii* [46, 47]. During *Brugia malayi* infection, NETs formation facilitated parasite attachment to leukocytes without inducing parasite death [48]. Further investigation into the characteristics of NETs released by PMNs in response to *E. cuniculi* would therefore be of particular interest. In particular, the presence of non-lytic NETs may trap parasites without causing their death, potentially decreasing the overall effectiveness of innate immune responses.

In the present study, we also observed that *E. cuniculi* parasites interacted with PMNs in different ways in response to host cell attacks. The increased expression of the β2 integrin CD11b reflected a slight activation of PMNs at early (1 h pi) and later (24 h pi) time points, although this activation remained much lower than that observed in Lipopolysaccharide (LPS)-stimulated PMNs used as a positive control [49]. At 24 h pi, considering the short half-life of PMNs, the activation of the remaining viable cells may be explained by the release of intracellular contents from dying cells, including spores, which may contribute to secondary activation. Consistent with these results, cytokine profile analysis did not reveal a strong inflammatory response in infected PMNs. In complementary experiments performed to assess the efficiency of phagocytosis, the proportion of *E. cuniculi* parasites associated with PMNs was unexpectedly about twofold lower (~ 5%) than that observed in non-phagocytic HFF cells (~ 10%) at 4 h pi (MOI 5, static conditions). Previous in vitro studies reported that approximately 60% and 35% of Vero cells were infected with *Encephalitozoon intestinalis* at 12 and 24 h pi, respectively (MOI 30, static conditions) [50]. Thus, phagocytic properties of target cells do not significantly impact the infection rates of *E. cuniculi*. One hypothesis could be inefficacy of spore recognition by PMNs. In general, pathogen-associated molecular patterns (PAMPs) associated with microsporidia are primarily recognized through the TLR pathways, particularly TLR2 and TLR4, which are known to recognize fungal agents and trigger an inflammatory response. For example, spore wall components such as GPI anchors, chitin, and spore wall proteins (SWPs) from *Encephalitozoon* species are recognized by TLR2, leading to activation of the NF-κB pathway and production of inflammatory mediators such as TNF-α and CXCL8 [51, 52]. Recognition of *E. cuniculi* PAMPs by PMNs through TLR2 or TLR4 could therefore occur. However, the surface expression levels of TLR2 and TLR4 are relatively low in PMNs compared with those observed in monocytes and MOs [53–55], which may limit efficient parasite recognition. Additionally, parasite-derived factors may interfere with PMN functions. For example, the spore wall protein SWP5 from *Nosema bombycis* was shown to protect spores from phagocytic uptake by non-immune insect-derived cells, suggesting a role in host–parasite interactions during infection [56]. Moreover, microsporidia may produce bioactive molecules that can modulate PMN functional properties. In particular, Bao et al. demonstrated that *Enterocytozoon hellem* secrete effectors such as the Ser/Thr phosphatase PP1, which interferes with the MAPK signaling pathway in DCs, thereby altering their immune functions. This results in reduced cytokine expression, impaired maturation, decreased phagocytic capacity, and diminished antigen-presenting ability [57]. Microsporidia can also actively invade eukaryotic cells using their unique and specialized invasion apparatus, the PT. The PT is tightly coiled within the spore and, once extruded, acts as a harpoon that pierces the host cell membrane to inject the sporoplasm into the host cytoplasm. Three main entry mechanisms, previously described for other host cell types [1, 7], were observed for the first time in this study in *E. cuniculi*-infected PMNs: (i) activated spores located near the target cell discharge their PT to penetrate the PMN membrane; (ii) intracellular spores discharge their PT within PMNs after phagocytosis to initiate infection; and (iii) adherent spores evaginate their PT to infect neighboring cells [23, 58–60]. To further explore these mechanisms, additional immunolabeling experiments could be performed to identify the PT proteins that specifically interact with the PMN membrane, as previously described in an *Anncaliia algerae* infection model [2]. To evaluate the relative proportion of parasites that enter inside PMNs through passive uptake (phagocytosis) versus active invasion via the PT, further experiments could be conducted using spores inactivated with paraformaldehyde (allowing phagocytosis only) or PMNs treated with cytochalasin D, a potent inhibitor of phagocytosis.

By exploring the different fates of *E. cuniculi* parasites within PMNs, we identified two different potential hacking strategies: intracellular development and/or silent transfer via PMN to other host cell types (e.g., MOs and fibroblasts). In vitro, the proportion of intracellular parasites increased over time, reaching nearly 10% at 48 h pi. This increase may be partly explained by the release of intracellular contents from dying cells, including spores, in the culture medium, leading to secondary uptake by PMN. Consequently, some intracellular spores detected at later time-points could have been recently phagocytosed. Nevertheless, intact CW^+^ spores were occasionally observed within PMNs at this time point. Ultrastructural analysis using transmission electron microscopy (TEM) confirmed the presence of intact spores within phagosomes. This suggests that *E. cuniculi* can evade degradation by lysosomal hydrolases within phagolysosomes, consistent with previous observations of infected PMNs during fish microsporidiosis [27] and in MOs [61]. Moreover, as described above, some intracellular spores were able to discharge their PT within PMNs after phagocytosis, potentially enabling sporoplasm injection and initiation of infection. Together, these results support the ability of some phagocytized spores to retain their functional properties within PMNs. Comparable results were obtained ex vivo, where intact spores maintaining their CW^+^ fluorescent signal were detected within PMNs purified from infected mouse ear pinna tissue. This observation is consistent with the persistence of the CW^+^ signal within EGFP^+^ phagocytes in the ear pinna tissue up to 72 h pi, as previously described [10]. Using FISH, we also observed parasite proliferation within PMNs for the first time. A small proportion of early developmental stages of *E. cuniculi* (meronts and sporonts) were detected within a parasitophorous vacuole (PV) by TEM. As previously described in other infected cell types (e.g., E6 and RK13 non-phagocytic cells), both meronts and sporonts displayed an ovoid morphology. Meronts were closely associated with the PV membrane, whereas sporonts were located in the center of the vacuole [62, 63]. These results illustrates, for the first time, the ability of *E. cuniculi* to initiate intracellular development within the highly microbicidal environment of PMNs, as previously reported in MOs [64, 65]. As minimalist parasites lacking essential genes for ATP production, *E. cuniculi* and other microsporidian species are known to recruit host mitochondria to the PV [22, 66]. In infected PMNs, mitochondria were observed in close proximity to the PV membrane, suggesting that the parasites may induce host mitochondrial remodeling to establish a favorable intracellular niche [50, 67]. Further investigation of the PV membrane in PMNs would be of particular interest, as previous studies have highlighted its unique properties, including the absence of canonical markers of endosomes, lysosomes, and the endoplasmic reticulum in fibroblastic L929 cells [65]. Additionally, parasite-derived effector molecules may contribute to adaptation to the hostile PMN environment and support intracellular survival and proliferation [66].

The last point addressed in this study concerns the role of intracellular intact spores or developing parasites in the outcome of *E. cuniculi* infection, in particular in parasite dissemination throughout the host. Recent research has demonstrated the ability of PMNs to leave the inflammatory site and to return to the bloodstream through a process known as reverse migration [68, 69]. Analysis of the inflammatory response at the inoculation site revealed a massive and sustained recruitment of EGFP+ phagocytes (MOs and PMNs) in the ear pinna model, from 2 h to 7 days pi. A proportion of these recruited cells harbored intracellular parasites at 24, 48 and 72 h pi [10]. In this context, PMNs containing viable intracellular parasites could potentially return to the bloodstream via reverse migration, thereby contributing to systemic dissemination of the infection. However, reverse migration is poorly characterized, and further investigation is needed to clarify this process [14]. Inflammatory cells, including PMN, are rapidly recruited to infected cutaneous tissue (as early as 2 h pi, present study). As discussed above, PMNs have a short lifespan and rapidly undergo cell death through multiple pathways, including non-lytic mechanisms such as apoptosis, as well as lytic processes such as NETosis, pyroptosis, necroptosis or ferroptosis (Tu et al., 2024). Consequently, two main fates can effectively be considered for infected PMNs harboring intracellular parasites (either spores or developing stages): (i) their uptake by other phagocytic cells, such as immature DCs or MOs (either tissue-resident or monocytes recruited from the bloodstream), which have a longer lifespan within tissues; or (ii) their lysis, leading to the release of their intracellular contents, including potentially viable parasites, into the extracellular environment. Efferocytosis, defined as the phagocytic clearance of apoptotic cells by other phagocytes, plays a central role in the resolution of inflammation [70, 71]. Efficient efferocytosis ensures the timely removal of apoptotic PMNs, preventing their progression to secondary necrosis and the subsequent release of teir toxic granule contents and damage-associated molecular patterns (DAMPs), which could otherwise amplify local inflammation and tissue injury [72]. However, several pathogens are known to hijack this process as a “Trojan Horse” mechanism, enabling their dissemination to distant tissues. In particular, apoptotic PMNs can serve as vehicles for intracellular pathogens, allowing them to survive and be transported away from the initial site of infection. This phenomenon has been described for parasites such as *L. major*,* L. donovani* [18, 25] and *T. gondii* [47]. In the context of microsporidiosis, efferocytosis of *E. cuniculi*-infected Jurkat cells by Bone Marrow-Derived Macrophages has been described in vitro [64]. Following uptake of infected apoptotic bodies, parasite developmental stages were detected within MOs, indicating successful intracellular survival and multiplication. Furthermore, *E. cuniculi* was shown to promote efferocytosis and to polarize MOs toward an M2-like phenotype, thereby creating a permissive environment for parasite persistence and supporting the hypothesis that MOs may act as “Trojan horses” [64]. In the present study, we observed the uptake of dying PMNs harboring intracellular *E. cuniculi* spores by MOs, likely through an efferocytosis-like mechanism, although all classical apoptotic features were not identified (fragmented aspect of nucleus, one typical morphological characteristic of apoptosis). Additional findings demonstrated that these intracellular spores retained their infectivity, as parasite development was observed in HFF cells co-incubated with sorted-infected PMNs. In separate experiments, a complete parasite life cycle was also observed within MOs. Taken together, these observations suggest that *E. cuniculi* may exploit PMNs as transient carriers of viable parasites, enabling their silent transfer to MOs. This mechanism is consistent with a Trojan horse-like strategy that could contribute to parasite dissemination and persistence within the host.

## Conclusions

This study provides new insights into the dynamic interactions between *E. cuniculi* microsporidia and PMN phagocytic cells, through the use of innovative and complementary in vitro and in vivo imaging approaches. While the efficiency of PMNs to eliminate parasite was only partial, as observed in MOs, we also found that *E. cuniculi* parasites can hijack PMNs to initiate their intracellular development and potentially use them as shuttles to enter silently inside MOs, therefore contributing to the spread of infection to other host target tissues. This process could have significant implications for the host adaptive immune response. Taken globally, these results contribute to a deeper understanding of how microsporidia manipulate the host immune system, particularly in terms of how the parasite may disseminate throughout the host organism.

## Supplementary Information

Additional file 1: Figure S1. Characteristics of the inoculum of *E*. *cuniculi* spores.

Additional file 2: Figure S2. Flow cytometry gating strategy for PMNs isolation from mouse bone marrow.

Additional files 3: Movies S1. Videomicroscopy of EGFP+ *E*. *cuniculi*-infected PMNs.

Additional file 4: Movie S2. Videomicroscopy of EGFP+ *E*. *cuniculi*-infected PMNs.

Additional file 5: Movie S3. Videomicroscopy of EGFP+ uninfected PMNs.

Additional file 6: Figure S3. Analysis of ellipticity prolate parameter for PMNs in contact with E. cuniculi spores.

.Additional file 7: Figure S4. Cytokine profile of PMNs infected with *E*. *cuniculi* spores.

Additional file 8: Figure S5. Analysis of PMNs interactions with *E*. *cuniculi* parasites.

Additional file 9: Figure S6. Flow cytometry analysis of PMN-parasite associations over time.

Additional file 10: Figure S7. E. cuniculi parasites initiate development inside PMNs in vitro.

Additional file 11: Figure S8. Development of *E*. *cuniculi* parasites inside HFF cells in vitro.

Additional file 12: Figure S9. *E*. *cuniculi* parasites induce a recruitment of inflammatory cells in vivo in the mouse ear pinna model.

Additional file 13: Figure S10. *E*. *cuniculi* parasites initiate development within PMNs in vivo.

Additional file 14: Figure S11. Entry of *E*. *cuniculi* parasites inside MOs Phagocytosis.

Additional file 15: Figure S12. *E*. *cuniculi* development inside HFF cells after co-incubation with dying PMNs harboring intracellular parasites.

Additional file 16: Supplementary Material and Methods.

## Data Availability

All data generated or analyzed during this study are included in this published article. The data sets generated and analyzed during the current study are available in the « BMC microbiology – Carriere et al.,» FigShare public repository website at https://figshare.com/s/2c0f289814b975f71684 .

## Abbreviations

CW +: Calcofluor White
DCs: Dendritic cells
*E. cuniculi*: *Encephalitozoon cuniculi*
FACS: Fluorescence-activated cell sorting
FISH: Fluorescence in situ Hybridization
HFF: Human Foreskin Fibroblast
*L. donovani*: *Leishmania donovani*
*L. major*: *Leishmania major*
LPS: Lipopolysaccharide
MGG: May-Grünwald-Giemsa
Me: Meronts
MOs: Macrophages
NETs: Neutrophil Extracellular Traps
PFA: Paraformaldehyde
PMA: Phorbol-12-Myristate-13-Acetate
PMNs: Neutrophils
PT: Polar tube
PV: Parasitophorous Vacuole
ROS: Reactive Oxygen Species
RT: Room Temperature
Spo: Sporont
*T. gondii*: *Toxoplasma gondii*
TEM: Transmission Electron Microscopy

## References

[1] Han Y, Gao H, Xu J, Luo J, Han B, Bao J, et al. Innate and Adaptive Immune Responses Against Microsporidia Infection in Mammals. Front Microbiol 26 juin. 2020;11. 10.3389/fmicb.2020.01468. 10.3389/fmicb.2020.01468 PMC7332555 32670257

[2] Fayet M, Long M, Han B, Belkorchia A, Delbac F, Polonais V. New insights into Microsporidia polar tube function and invasion mechanism. J Eukaryot Microbiol. 2024;71(5):e13043. 10.1111/jeu.13043. 38973152 10.1111/jeu.13043

[3] Han B, Weiss LM. Microsporidia: Obligate Intracellular Pathogens Within the Fungal Kingdom. Heitman J, Stukenbrock EH, éditeurs. Microbiol Spectr. 10 mars 2017;5(2):5.2.03. doi:10.1128/microbiolspec.FUNK-0018-2016 10.1128/microbiolspec.funk-0018-2016 PMC5613672 28944750

[4] Han B, Pan G, Weiss LM. Microsporidiosis in Humans. Clin Microbiol Rev 15 déc. 2021;34(4):e00010–20. 10.1128/CMR.00010-20. 10.1128/CMR.00010-20 PMC8404701 34190570

[5] Ghosh K, Weiss LM. T cell response and persistence of the microsporidia. FEMS Microbiol Rev 1 mai. 2012;36(3):748–60. 10.1111/j.1574-6976.2011.00318.x. 10.1111/j.1574-6976.2011.00318.x 22126330

[6] Nourrisson C, Lavergne RA, Moniot M, Morio F, Poirier P. Enterocytozoon bieneusi, a human pathogen. Emerg Microbes Infect 31 déc. 2024;13(1):2406276. 10.1080/22221751.2024.2406276. PubMed PMID: 39286988. 10.1080/22221751.2024.2406276 PMC11428314 39286988

[7] Valenčáková A, Halánová M, Bálent P, Dvorožnáková E, Jamborová E, Lešník F, et al. Immune response in mice infected by Encephalitozoon cuniculi and suppressed by dexamethasone. Acta Vet Hung 22 juill. 2005;52(1):61–9. 10.1556/avet.52.2004.1.7. 10.1556/AVet.52.2004.1.7 15119788

[8] van Gool T, Vetter JC, Weinmayr B, Van Dam A, Derouin F, Dankert J. High seroprevalence of Encephalitozoon species in immunocompetent subjects. J Infect Dis avr. 1997;175(4):1020–4. doi:10.1086/513963 PubMed PMID: 9086174. 10.1086/513963 9086174

[9] Ruan Y, Xu X, He Q, Li L, Guo J, Bao J, et al. The largest meta-analysis on the global prevalence of microsporidia in mammals, avian and water provides insights into the epidemic features of these ubiquitous pathogens. Parasit Vectors 1 avr. 2021;14(1):186. 10.1186/s13071-021-04700-x. 10.1186/s13071-021-04700-x PMC8017775 33794979

[10] Carriere E, Abdul Hamid AI, Feki I, Dubuffet A, Delbac F, Gueirard P. A mouse ear skin model to study the dynamics of innate immune responses against the microsporidian Encephalitozoon cuniculi. Front Microbiol 13 avr. 2023;14. 10.3389/fmicb.2023.1168970. 10.3389/fmicb.2023.1168970 PMC10136781 37125152

[11] Mathews A, Hotard A, Hale-Donze H. Innate immune responses to Encephalitozoon species infections. Microbes Infect oct. 2009;11(12):905–11. 10.1016/j.micinf.2009.06. .004 PubMed PMID: 19573618. 10.1016/j.micinf.2009.06.004 19573618

[12] Brdíčková K, Sak B, Holubová N, Květoňová D, Hlásková L, Kicia M, et al. Encephalitozoon cuniculi Genotype II Concentrates in Inflammation Foci. J Inflamm Res 25 sept. 2020;13:583–93. 10.2147/JIR.S271628. 10.2147/JIR.S271628 PMC7524191 33061524

[13] Sak B, Holubová N, Květoňová D, Hlásková L, Tinavská J, Kicia M et al. Comparison of the Concentration of Encephalitozoon cuniculi Genotypes I and III in Inflammatory Foci Under Experimental Conditions. J Inflamm Res avr 2022;Volume 15:2721–30. 10.2147/JIR.S363509 10.2147/JIR.S363509 PMC9056047 35502243

[14] Burn GL, Foti A, Marsman G, Patel DF, Zychlinsky A. Neutrophil Immun 13 juill. 2021;54(7):1377–91. 10.1016/j.immuni.2021.06.006. 10.1016/j.immuni.2021.06.006 34260886

[15] Malech HL, DeLeo FR, Quinn MT. The Role of Neutrophils in the Immune System: An Overview. Methods Mol Biol Clifton NJ. 2014;1124:3–10. 10.1007/978-1. -62703-845-4_1 PubMed PMID: 24504942; PubMed Central PMCID: PMC6777345. 10.1007/978-1-62703-845-4_1 PMC6777345 24504942

[16] Nicolás-Ávila JÁ, Adrover JM, Hidalgo A. Neutrophils in Homeostasis, Immunity, and Cancer. Immun 17 janv. 2017;46(1):15–28. 10.1016/j.immuni.2016.12. .012 PubMed PMID: 28099862. 10.1016/j.immuni.2016.12.012 28099862

[17] Dubuffet A, Smith JE, Solter L, Perotti MA, Braig HR, Dunn AM. Specific Detection and Localization of Microsporidian Parasites in Invertebrate Hosts by Using In Situ Hybridization. Appl Environ Microbiol janv. 2013;79(1):385–8. 10.1128/AEM.02699-12. 10.1128/AEM.02699-12 PMC3536083 23087031

[18] Gueirard P, Laplante A, Rondeau C, Milon G, Desjardins M. Trafficking of Leishmania donovani promastigotes in non-lytic compartments in neutrophils enables the subsequent transfer of parasites to macrophages. Cell Microbiol janv. 2008;10(1):100–11. 10.1111/j.1462-5822.2007.01018. .x PubMed PMID: 17651446. 10.1111/j.1462-5822.2007.01018.x 17651446

[19] Kim M, Lu RJ, Benayoun BA. Single-cell RNA-seq of primary bone marrow neutrophils from female and male adult mice. Sci Data 23 juill. 2022;9:442. 10.1038/s41597-022-01544-7. PubMed PMID: 35871169; PubMed Central PMCID: PMC9308797. 10.1038/s41597-022-01544-7 PMC9308797 35871169

[20] Toda G, Yamauchi T, Kadowaki T, Ueki K. Preparation and culture of bone marrow-derived macrophages from mice for functional analysis. STAR Protoc mars. 2021;2(1):100246. 10.1016/j.xpro.2020.100246. 10.1016/j.xpro.2020.100246 PMC7797923 33458708

[21] Abdul Hamid AI, Cara A, Diot A, Laurent F, Josse J, Gueirard P. Differential Early in vivo Dynamics and Functionality of Recruited Polymorphonuclear Neutrophils After Infection by Planktonic or Biofilm Staphylococcus aureus. Front Microbiol 30 août. 2021;12. 10.3389/fmicb.2021.728429. 10.3389/fmicb.2021.728429 PMC8435793 34526981

[22] Texier C, Vidau C, Viguès B, El Alaoui H, Delbac F. Microsporidia: a model for minimal parasite–host interactions. Curr Opin Microbiol août. 2010;13(4):443–9. 10.1016/j.mib.2010.05.005. 10.1016/j.mib.2010.05.005 20542726

[23] Delbac F, Polonais V. The Microsporidian Polar Tube and Its Role in Invasion. In: Burleigh BA, Soldati-Favre D, éditeurs. Molecular Mechanisms of Parasite Invasion [Internet]. New York, NY: Springer New York; 2008 [cité 7 févr 2025]. pp. 208–20. (Subcellular Biochemistry). Disponible sur: http://link.springer.com/10.1007/978-0-387-78267-6_17 doi:10.1007/978-0-387-78267-6_17.

[24] Savill J, Fadok V, Henson P, Haslett C. Phagocyte recognition of cells undergoing apoptosis. Immunol Today 1 janv. 1993;14(3):131–6. 10.1016/0167-5699(93)90215-7. 10.1016/0167-5699(93)90215-7 8385467

[25] van Zandbergen G, Klinger M, Mueller A, Dannenberg S, Gebert A, Solbach W, et al. Cutting Edge: Neutrophil Granulocyte Serves as a Vector for Leishmania Entry into Macrophages1. J Immunol 1 déc. 2004;173(11):6521–5. 10.4049/jimmunol.173.11.6521. 10.4049/jimmunol.173.11.6521 15557140

[26] Weber R, Bryan RT, Schwartz DA, Owen RL. Human Microsporidial Infections. CLIN MICROBIOL REV. 1994. 10.1128/cmr.7.4.426 PMC358336 7834600

[27] Rodriguez-Tovar LE, Speare DJ, Markham RJF. Fish microsporidia: Immune response, immunomodulation and vaccination. Fish Shellfish Immunol avr. 2011;30(4–5):999–1006. 10.1016/j.fsi.2011.02.011. 10.1016/j.fsi.2011.02.011 21352922

[28] Alakesh A, Jothiprakasam T, Raghavan JV, Jhunjhunwala S. Sterile inflammation alters neutrophil kinetics in mice. J Leukoc Biol 24 août. 2022;112(3):395–409. 10.1002/JLB.1A0321-132RR. 10.1002/JLB.1A0321-132RR PMC7616002 35172385

[29] Deniset JF, Kubes P. Neutrophil heterogeneity: Bona fide subsets or polarization states? J Leukoc Biol 7 mai. 2018;103(5):829–38. 10.1002/JLB.3RI0917-361R. 10.1002/JLB.3RI0917-361R 29462505

[30] Fan Y, Teng Y, Liu F, tong, Ma F, Hsu AY, Feng S, et al. Neutrophil Lifespan Extension with CLON-G and an In Vitro Spontaneous Death Assay. J Vis Exp JoVE 12 mai. 2023;195e65132. 10.3791/65132. 10.3791/65132 37246861

[31] Fritz-Laylin LK, Riel-Mehan M, Chen BC, Lord SJ, Goddard TD, Ferrin TE, et al. Actin-based protrusions of migrating neutrophils are intrinsically lamellar and facilitate direction changes. eLife. 2017;6:e26990. 10. 7554/eLife.26990 PubMed PMID: 28948912; PubMed Central PMCID: PMC5614560. 28948912 10.7554/eLife.26990 PMC5614560

[32] Mejillano MR, Kojima S, ichiro, Applewhite DA, Gertler FB, Svitkina TM, Borisy GG. Lamellipodial Versus Filopodial Mode of the Actin Nanomachinery: Pivotal Role of the Filament Barbed End. Cell 6 août. 2004;118(3):363–73. 10.1016/j.cell.2004.07.019. 10.1016/j.cell.2004.07.019 15294161

[33] Mylvaganam S, Freeman SA, Grinstein S. The cytoskeleton in phagocytosis and macropinocytosis. Curr Biol 24 mai. 2021;31(10):R619–32. 10.1016/j.cub.2021.01.036. 10.1016/j.cub.2021.01.036 34033794

[34] Svitkina TM. Filopodia and Lamellipodia. In: Encyclopedia of Cell Biology [Internet]. Elsevier; 2016 [cité 12 févr 2025]. pp. 683–93.

[35] You R, Li X, Liu Y, Liu G, Lu S, Li M. Response of filopodia and lamellipodia to surface topography on micropatterned silk fibroin films. J Biomed Mater Res déc. 2014;102(12):4206–12. 10.1002/jbm.a.35097. PubMed PMID: 24464986. 10.1002/jbm.a.35097 24464986

[36] Fites JS, Gui M, Kernien JF, Negoro P, Dagher Z, Sykes DB, et al. An unappreciated role for neutrophil-DC hybrids in immunity to invasive fungal infections. PLoS Pathog 21 mai. 2018;14(5):e1007073. 10.1371/journal.ppat.1007073. PubMed PMID: 29782541; PubMed Central PMCID: PMC5983859. 10.1371/journal.ppat.1007073 PMC5983859 29782541

[37] Rocheleau AD, Sumagin R, Sarelius IH, King MR. Simulation and Analysis of Tethering Behavior of Neutrophils with Pseudopods. PLoS ONE. 19 juin. 2015;10(6):e0128378. 10.1371/journal.pone.0128378 PubMed PMID: 26091091; PubMed Central PMCID: PMC4474963. 10.1371/journal.pone.0128378 PMC4474963 26091091

[38] Roberts RE, Hallett MB. Neutrophil Cell Shape Change: Mechanism and Signalling during Cell Spreading and Phagocytosis. Int J Mol Sci 19 mars. 2019;20(6):1383. 10.3390/ijms20061383. PubMed PMID: 30893856; PubMed Central PMCID: PMC6471475. 10.3390/ijms20061383 PMC6471475 30893856

[39] Lekkala S, Ren Y, Weeks J, Lee K, Tay AJH, Liu B, et al. A semi-automated cell tracking protocol for quantitative analyses of neutrophil swarming to sterile and S. aureus contaminated bone implants in a mouse femur model. PLOS ONE 20 juin. 2024;19(6):e0296140. 10.1371/journal.pone.0296140. 10.1371/journal.pone.0296140 PMC11189170 38900759

[40] Nordenfelt P, Tapper H. Phagosome dynamics during phagocytosis by neutrophils. J Leukoc Biol 19 avr. 2011;90(2):271–84. 10.1189/jlb.0810457. 10.1189/jlb.0810457 21504950

[41] Lee WL, Harrison RE, Grinstein S. Phagocytosis by neutrophils. Microbes Infect nov. 2003;5(14):1299–306. 10.1016/j.micinf.2003.09.014. 10.1016/j.micinf.2003.09.014 14613773

[42] Rosazza T, Warner J, Sollberger G. NET formation – mechanisms and how they relate to other cell death pathways. FEBS J juin. 2021;288(11):3334–50. 10.1111/febs.15589. 10.1111/febs.15589 33047496

[43] Wang H, Kim SJ, Lei Y, Wang S, Wang H, Huang H, et al. Neutrophil extracellular traps in homeostasis and disease. Signal Transduct Target Ther 20 sept. 2024;9(1):235. 10.1038/s41392-024-01933-x. 10.1038/s41392-024-01933-x PMC11415080 39300084

[44] Demkow U. Molecular Mechanisms of Neutrophil Extracellular Trap (NETs) Degradation. Int J Mol Sci 3 mars. 2023;24(5):4896. 10.3390/ijms24054896. 10.3390/ijms24054896 PMC10002918 36902325

[45] Kapoor D, Shukla D. Neutrophil Extracellular Traps and Their Possible Implications in Ocular Herpes Infection. Pathogens 29 janv. 2023;12(2):209. 10.3390/pathogens12020209. 10.3390/pathogens12020209 PMC9958879 36839481

[46] Firouzjaie F, Taghipour N, Akhavan AA, Seyyed Tabaei SJ, Rouhani S, Shirazian M, et al. Neutrophil extracellular traps formation: effect of Leishmania major promastigotes and salivary gland homogenates of Phlebotomus papatasi in human neutrophil culture. BMC Microbiol 4 avr. 2024;24(1):117. 10.1186/s12866-024-03270-z. 10.1186/s12866-024-03270-z PMC10993452 38575882

[47] Macedo IS, Lara FA, Barbosa HS, Saraiva EM, Menna-Barreto RFS, Mariante RM. Human neutrophil extracellular traps do not impair in vitro Toxoplasma gondii infection. Front Immunol 5 déc. 2023;14:1282278. 10.3389/fimmu.2023.1282278. PubMed PMID: 38115994; PubMed Central PMCID: PMC10728484. 10.3389/fimmu.2023.1282278 PMC10728484 38115994

[48] McCoy CJ, Reaves BJ, Giguère S, Coates R, Rada B, Wolstenholme AJ. Human Leukocytes Kill Brugia malayi Microfilariae Independently of DNA-Based Extracellular Trap Release. PLoS Negl Trop Dis 3 janv. 2017;11(1):e0005279. 10.1371/journal.pntd.0005279. 10.1371/journal.pntd.0005279 PMC5234842 28045905

[49] Weirich E, Rabin RL, Maldonado Y, Benitz W, Modler S, Herzenberg LA, et al. Neutrophil CD11b expression as a diagnostic marker for early-onset neonatal infection. J Pediatr 1 mars. 1998;132(3):445–51. 10.1016/S0022-3476(98)70018-6. 10.1016/s0022-3476(98)70018-6 9544899

[50] Antao NV, Lam C, Davydov A, Riggi M, Sall J, Petzold C, et al. 3D reconstructions of parasite development and the intracellular niche of the microsporidian pathogen Encephalitozoon intestinalis. Nat Commun 23 nov. 2023;14:7662. 10.1038/s41467-023-43215-0. PubMed PMID: 37996434; PubMed Central PMCID: PMC10667486. 10.1038/s41467-023-43215-0 PMC10667486 37996434

[51] Fischer J, Suire C, Hale-Donze H. Toll-Like Receptor 2 Recognition of the Microsporidia Encephalitozoon spp. Induces Nuclear Translocation of NF-κB and Subsequent Inflammatory Responses. Infect Immun oct. 2008;76(10):4737–44. 10.1128/IAI.00733-08. 10.1128/IAI.00733-08 PMC2546815 18678660

[52] Lawlor EM, Moretto MM, Khan IA. Optimal CD8 T-Cell Response against *Encephalitozoon cuniculi* Is Mediated by Toll-Like Receptor 4 Upregulation by Dendritic Cells. Infect Immun juill. 2010;78(7):3097–102. 10.1128/IAI.00181-10. 10.1128/IAI.00181-10 PMC2897398 20421379

[53] Zhang R, Zheng W, Daugschies A, Bangoura B. Apicomplexan co-infections impair with phagocytic activity in avian macrophages. Parasitol Res déc. 2020;119(12):4159–68. 10.1007/s00436-020-06900-3. 10.1007/s00436-020-06900-3 PMC7704517 33029719

[54] Parker LC, Jones EC, Prince LR, Dower SK, Whyte MKB, Sabroe I. Endotoxin tolerance induces selective alterations in neutrophil function. J Leukoc Biol 21 oct. 2005;78(6):1301–5. 10.1189/jlb.0405236. 10.1189/jlb.0405236 16244113

[55] Prince LR, Allen L, Jones EC, Hellewell PG, Dower SK, Whyte MKB et al. The Role of Interleukin-1 in Direct and Toll-Like Receptor 4-Mediated Neutrophil Activation and Survival. 165. 2004;165(5). 10.1016/s0002-9440(10)63437-2 PMC1618681 15509550

[56] Cai S, Lu X, Qiu H, Li M, Feng Z. Identification of a Nosema bombycis (Microsporidia) spore wall protein corresponding to spore phagocytosis. Parasitol août. 2011;138(9):1102–9. 10.1017/S0031182011000801. 10.1017/S0031182011000801 21756420

[57] Bao J, Tang Y, Chen Y, Jin J, Wang X, An G, et al. E. hellem Ser/Thr protein phosphatase PP1 targets the DC MAPK pathway and impairs immune functions. Life Sci Alliance avr. 2024;7(4):e202302375. 10.26508/lsa.202302375. 10.26508/lsa.202302375 PMC10781585 38199846

[58] Franzen C. How do microsporidia invade cells? Folia Parasitol (Praha). 1 mai. 2005;52(1–2):36–40. 10.14411/fp.2005.005. 10.14411/fp.2005.005 16004362

[59] Franzen C. Microsporidia: how can they invade other cells? Trends Parasitol. juin. 2004;20(6):275–9. 10.1016/j.pt.2004.04.009 10.1016/j.pt.2004.04.009 15147678

[60] Takvorian PM, Buttle KF, Mankus D, Mannella CA, Weiss LM, Cali A. The Multilayered Interlaced Network (MIN) in the sporoplasm of the Microsporidium nncaliia algerae is derived from Golgi. J Eukaryot Microbiol. 2013;60(2):166–78. 10.1111/jeu.12019. 23316714 10.1111/jeu.12019 PMC3751416

[61] Pereira A, Alvares-Saraiva AM, Konno FT, de C, Spadacci-Morena DD, Perez EC, Mariano M, et al. B-1 cell-mediated modulation of M1 macrophage profile ameliorates microbicidal functions and disrupt the evasion mechanisms of Encephalitozoon cuniculi. PLoS Negl Trop Dis 19 sept. 2019;13(9):e0007674. 10.1371/journal.pntd.0007674. PubMed PMID: 31536488; PubMed Central PMCID: PMC6779274. 10.1371/journal.pntd.0007674 PMC6779274 31536488

[62] Bohne W, Böttcher K, Gross U. The parasitophorous vacuole of Encephalitozoon cuniculi: biogenesis and characteristics of the host cell-pathogen interface. Int J Med Microbiol IJMM juin. 2011;301(5):395–9. 10.1016/j.ijmm.2011.04.006. PubMed PMID: 21550847. 10.1016/j.ijmm.2011.04.006 21550847

[63] Hacker C, Howell M, Bhella D, Lucocq J. Strategies for maximizing ATP supply in the microsporidian Encephalitozoon cuniculi: direct binding of mitochondria to the parasitophorous vacuole and clustering of the mitochondrial porin VDAC. Cell Microbiol déc. 2013;16(4):565–79. 10.1111/cmi.12240. PubMed PMID: 24245785; PubMed Central PMCID: PMC4233961. 10.1111/cmi.12240 PMC4233961 24245785

[64] Dalboni LC, Saraiva AMA, Konno FT, de Perez C, Codeceira EC, Spadacci-Morena JF. Encephalitozoon cuniculi takes advantage of efferocytosis to evade the immune response. PLOS ONE 5 mars. 2021;16(3):e0247658. 10.1371/journal.pone.0247658. 10.1371/journal.pone.0247658 PMC7935246 33667240

[65] Fasshauer V, Gross U, Bohne W. The Parasitophorous Vacuole Membrane of Encephalitozoon cuniculi Lacks Host Cell Membrane Proteins Immediately after Invasion. Eukaryot Cell janv. 2005;4(1):221–4. 10.1128/EC.4.1.221-224.2005. 10.1128/EC.4.1.221-224.2005 PMC544160 15643077

[66] Ran M, Yang W, Faryad Khan MU, Li T, Pan G. Microsporidia secretory effectors and their roles in pathogenesis. J Eukaryot Microbiol. 2024;71(5):e13046. 10.1111/jeu.13046. PubMed PMID: 39228342. 39228342 10.1111/jeu.13046

[67] Zhou M, Yang Z, Zhang X, Zhu Z, Xu Y, Xin Z, et al. Ameson portunus infection is associated with RK13 mitochondrial abnormalities. J Invertebr Pathol sept. 2025;212:108390. 10.1016/j.jip.2025.108390. 10.1016/j.jip.2025.108390 40543896

[68] Mathias JR, Perrin BJ, Liu TX, Kanki J, Look AT, Huttenlocher A. Resolution of inflammation by retrograde chemotaxis of neutrophils in transgenic zebrafish. J Leukoc Biol déc. 2006;80(6):1281–8. 10.1189/jlb.0506346. PubMed PMID: 16963624. 10.1189/jlb.0506346 16963624

[69] Giese MA, Hind LE, Huttenlocher A. Neutrophil plasticity in the tumor microenvironment. Blood 16 mai. 2019;133(20):2159–67. 10.1182/blood-2018-11-844548. 10.1182/blood-2018-11-844548 PMC6524564 30898857

[70] Ren Y, Stuart L, Lindberg FP, Rosenkranz AR, Chen Y, Mayadas TN, et al. Nonphlogistic Clearance of Late Apoptotic Neutrophils by Macrophages: Efficient Phagocytosis Independent of β2 Integrins1. J Immunol 1 avr. 2001;166(7):4743–50. 10.4049/jimmunol.166.7.4743. 10.4049/jimmunol.166.7.4743 11254736

[71] Stark MA, Huo Y, Burcin TL, Morris MA, Olson TS, Ley K. Phagocytosis of apoptotic neutrophils regulates granulopoiesis via IL-23 and IL-17. Immun mars. 2005;22(3):285–94. 10.1016/j.immuni.2005.01.011. PubMed PMID: 15780986. 10.1016/j.immuni.2005.01.011 15780986

[72] Tu H, Ren H, Jiang J, Shao C, Shi Y, Li P. Dying to Defend: Neutrophil Death Pathways and their Implications in Immunity. Adv Sci févr. 2024;11(8):2306457. 10.1002/advs.202306457. 10.1002/advs.202306457 PMC10885667 38044275

