# Supplementary figures and images for: llluminating the live-cell dynamics of early interactions between neutrophils and the microsporidian parasite Encephalitozoon cuniculi
Source: BMC Microbiol. 2026 May 11;26:587. doi: 10.1186/s12866-026-04989-7 (PMC13330375; doi:10.1186/s12866-026-04989-7)

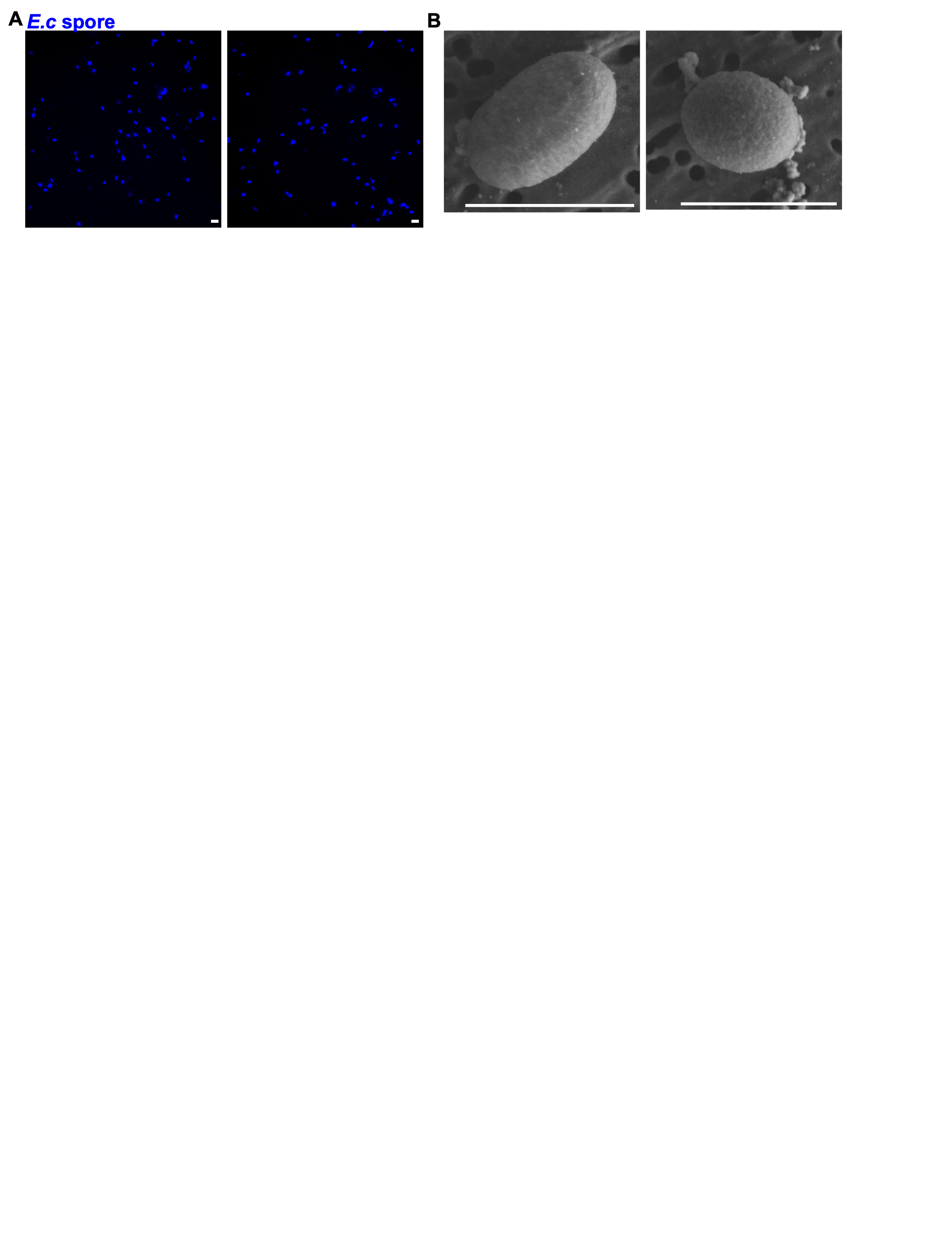

Supplement: Supplementary file 1 — Additional file 1: Figure S1. Characteristics of the inoculum of E. cuniculi spores. [file 12866_2026_4989_MOESM1_ESM.tiff]

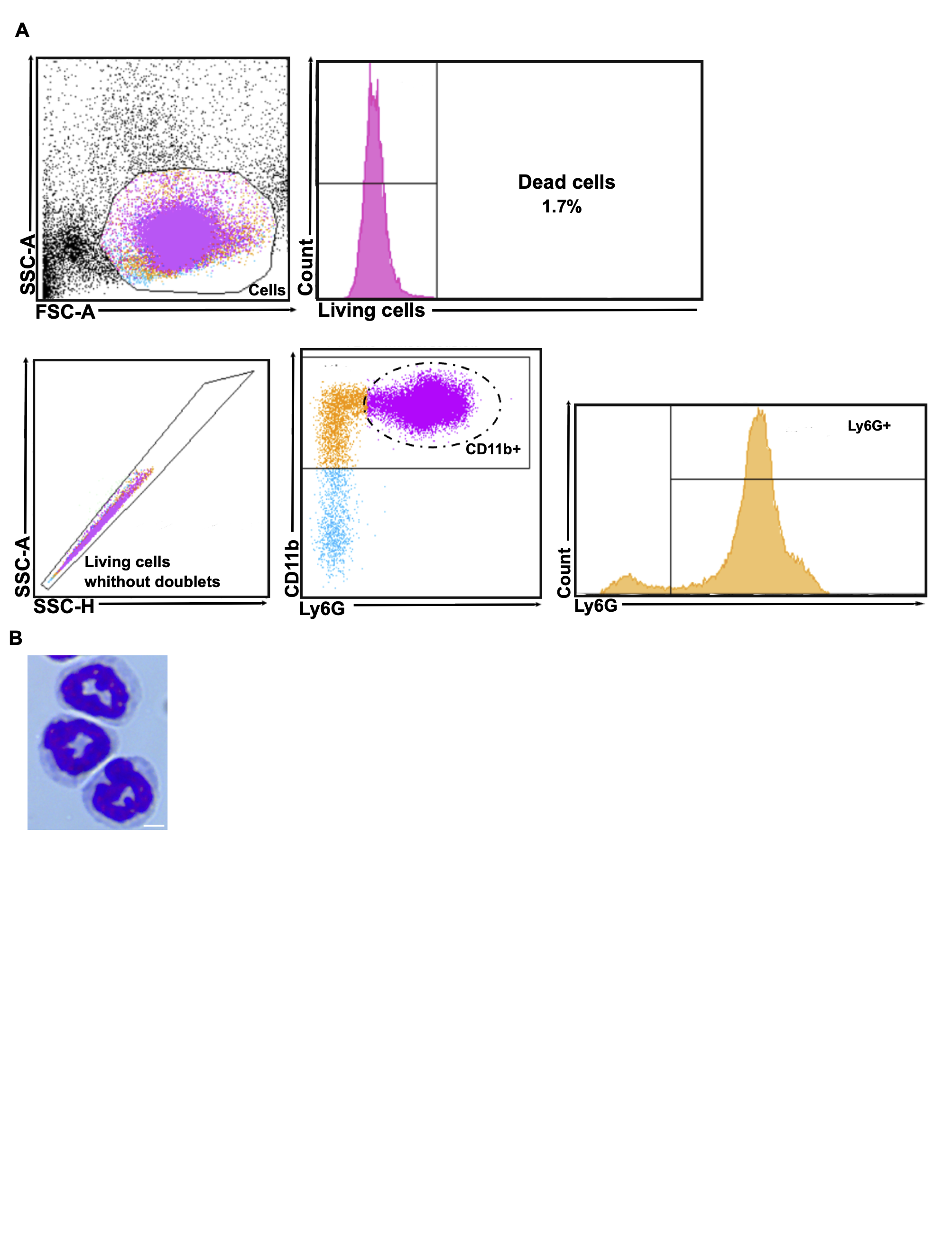

Supplement: Supplementary file 2 — Additional file 2: Figure S2. Flow cytometry gating strategy for PMNs isolation from mouse bone marrow. [file 12866_2026_4989_MOESM2_ESM.tiff]

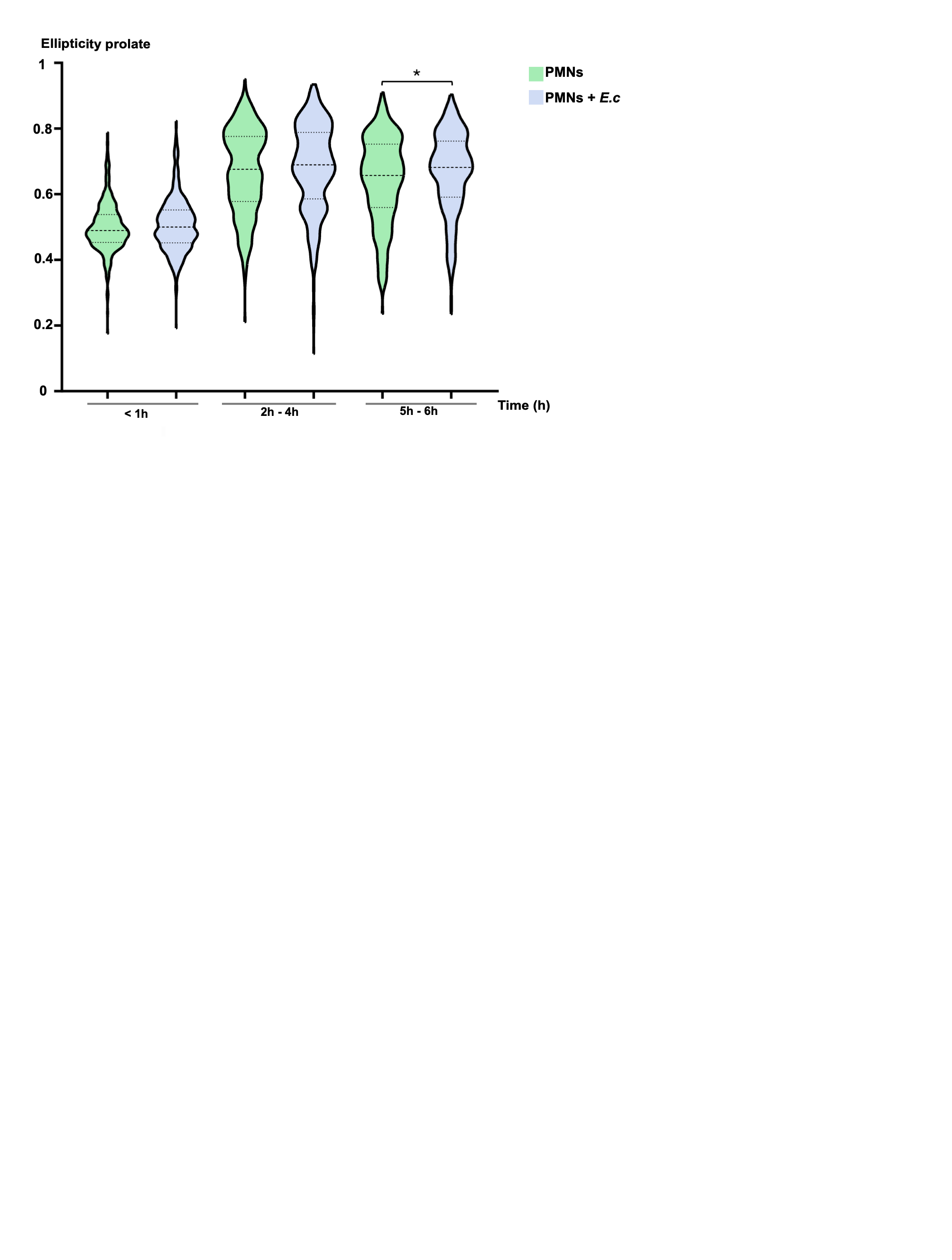

Supplement: Supplementary file 6 — Additional file 6: Figure S3. Analysis of ellipticity prolate parameter for PMNs in contact with E. cuniculi spores. [file 12866_2026_4989_MOESM6_ESM.tiff]

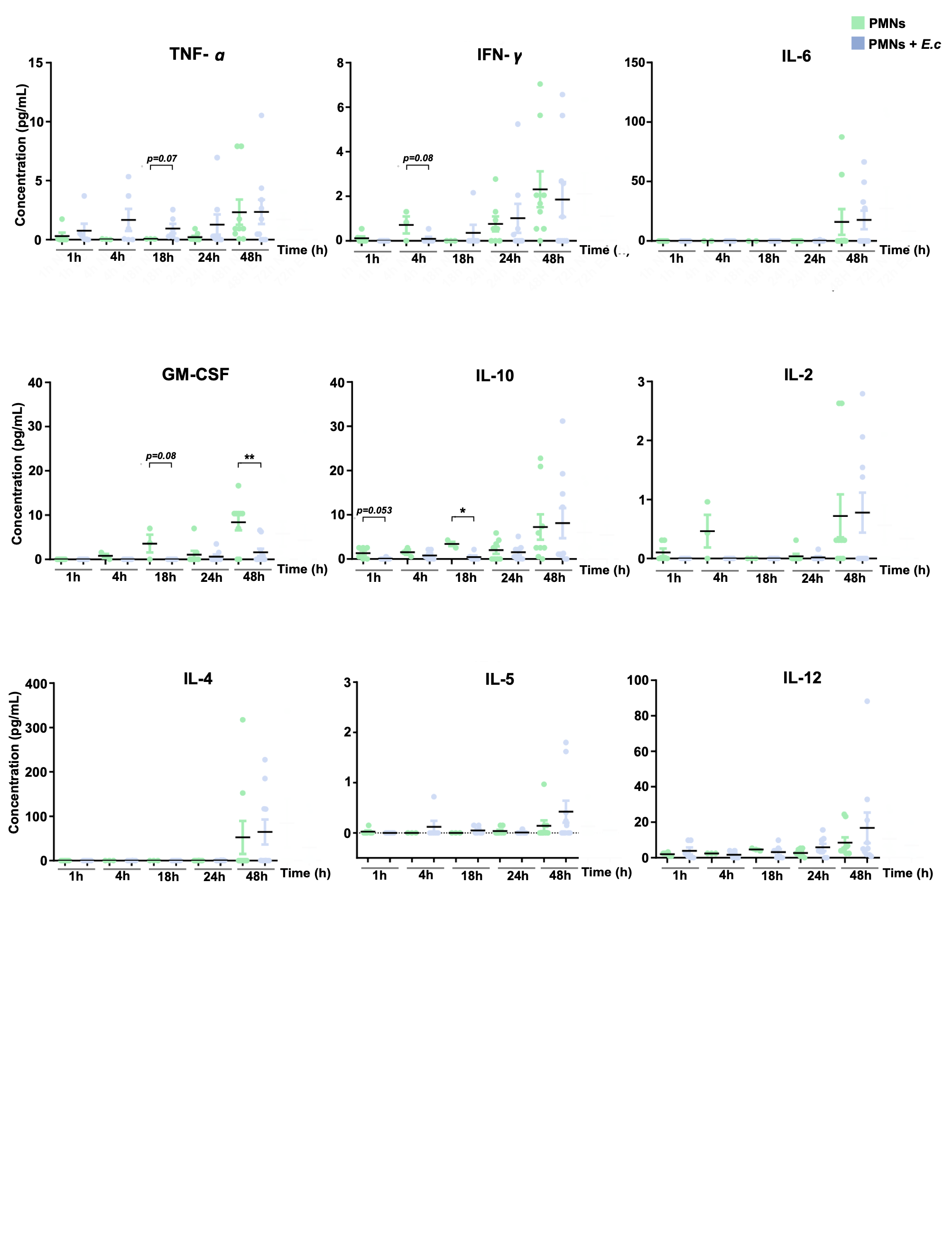

Supplement: Supplementary file 7 — .Additional file 7: Figure S4. Cytokine profile of PMNs infected with E. cuniculi spores. [file 12866_2026_4989_MOESM7_ESM.tiff]

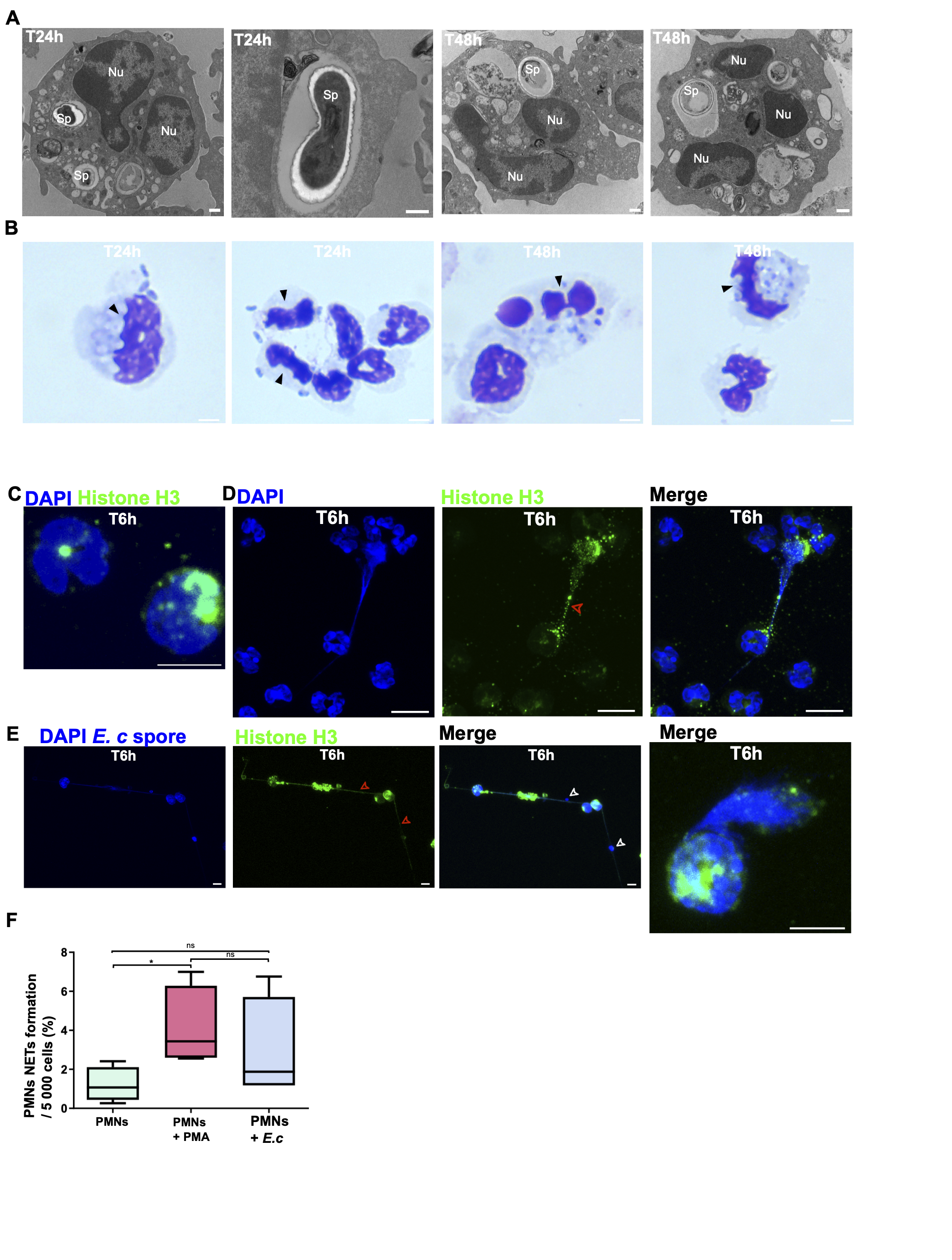

Supplement: Supplementary file 8 — Additional file 8: Figure S5. Analysis of PMNs interactions with E. cuniculi parasites. [file 12866_2026_4989_MOESM8_ESM.tiff]

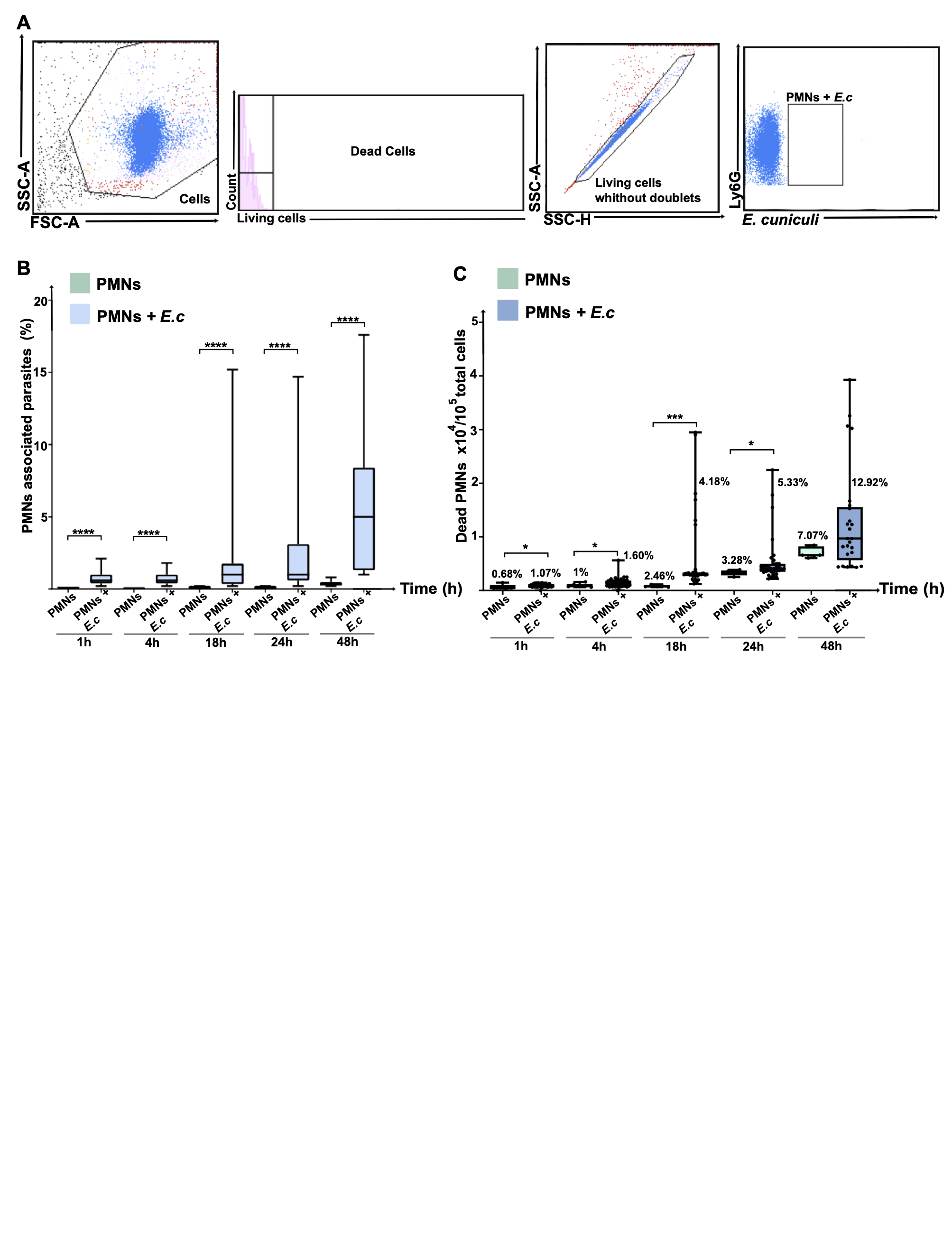

Supplement: Supplementary file 9 — Additional file 9: Figure S6. Flow cytometry analysis of PMN-parasite associations over time. [file 12866_2026_4989_MOESM9_ESM.tiff]

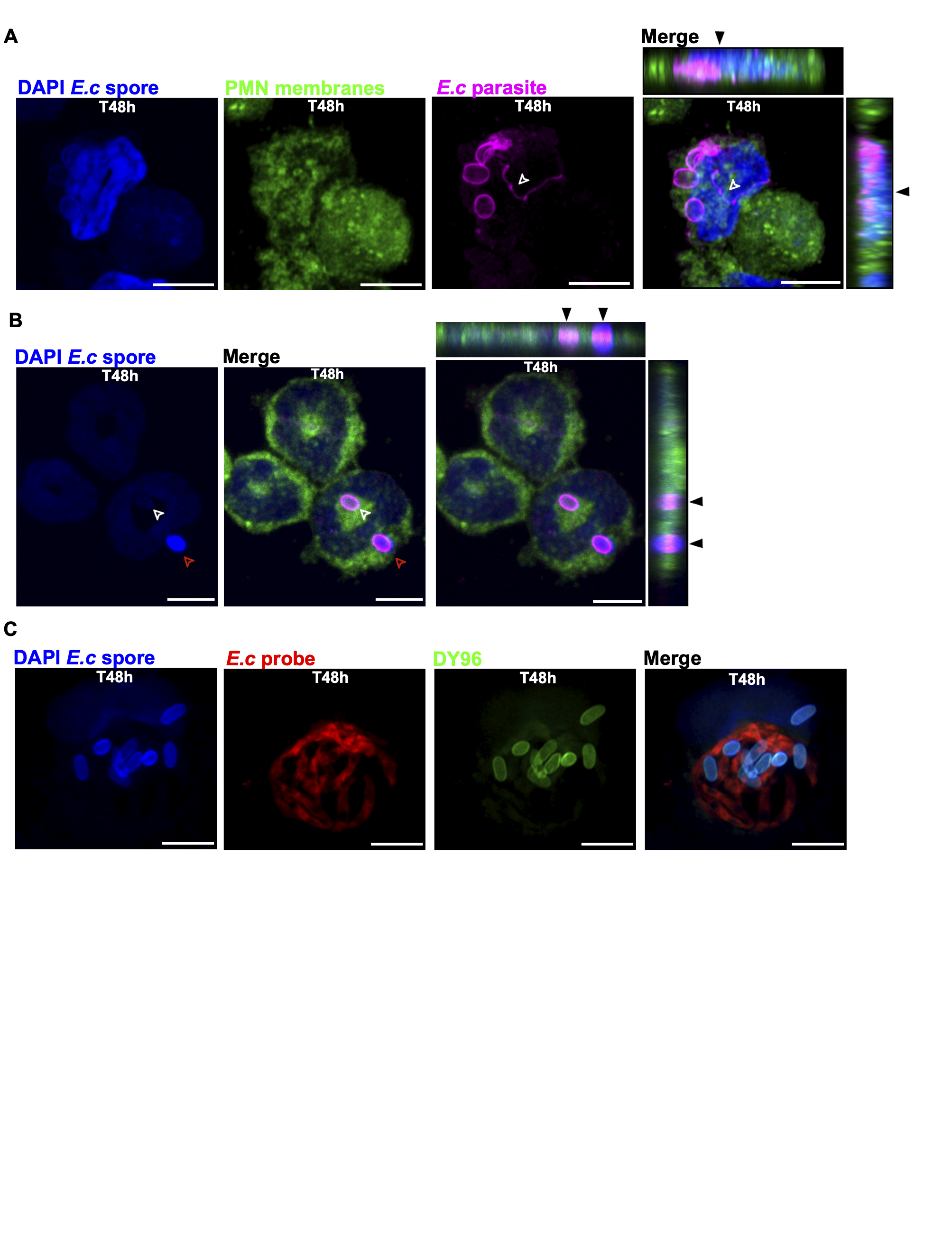

Supplement: Supplementary file 10 — Additional file 10: Figure S7. E. cuniculi parasites initiate development inside PMNs in vitro. [file 12866_2026_4989_MOESM10_ESM.tiff]

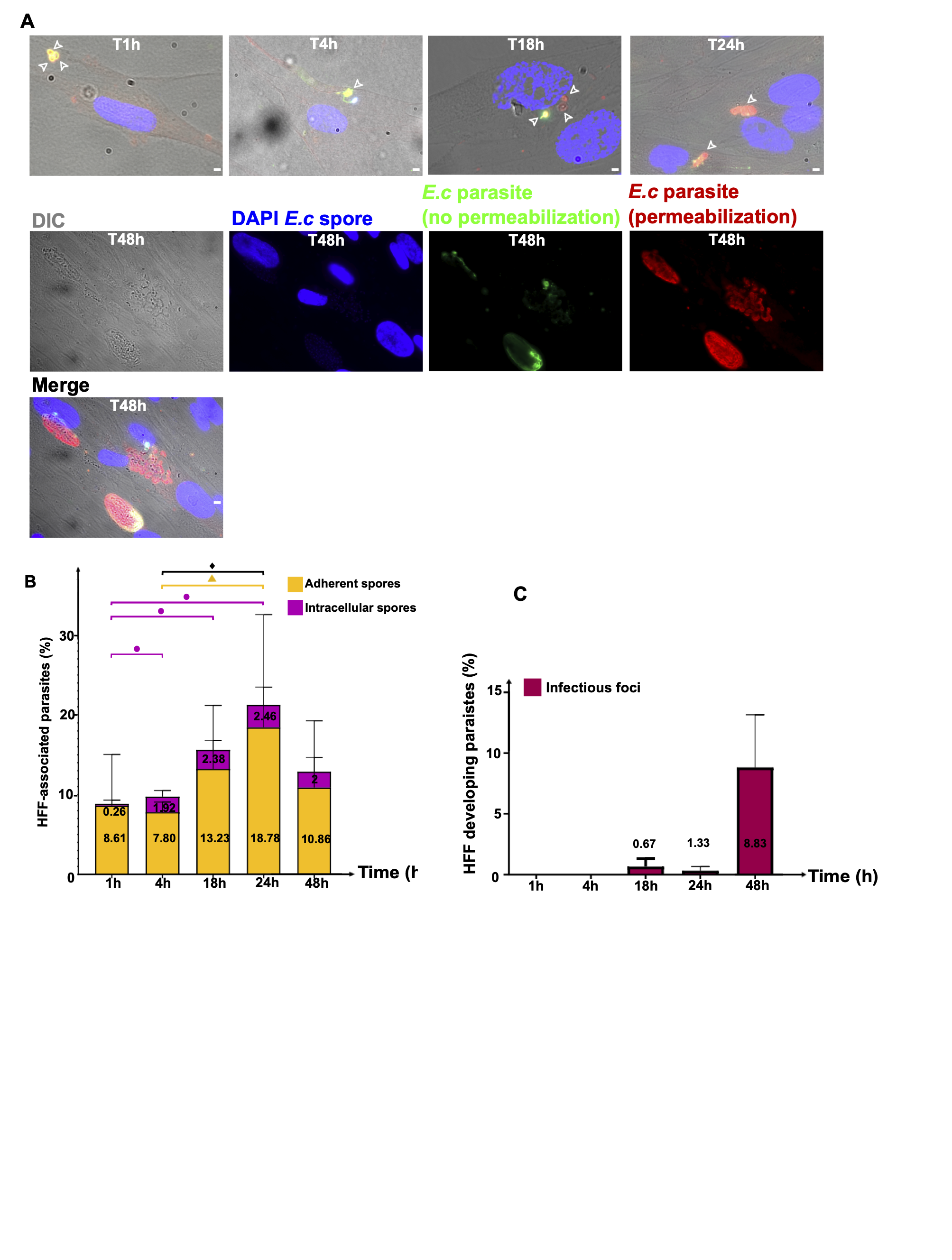

Supplement: Supplementary file 11 — Additional file 11: Figure S8. Development of E. cuniculi parasites inside HFF cells in vitro. [file 12866_2026_4989_MOESM11_ESM.tiff]

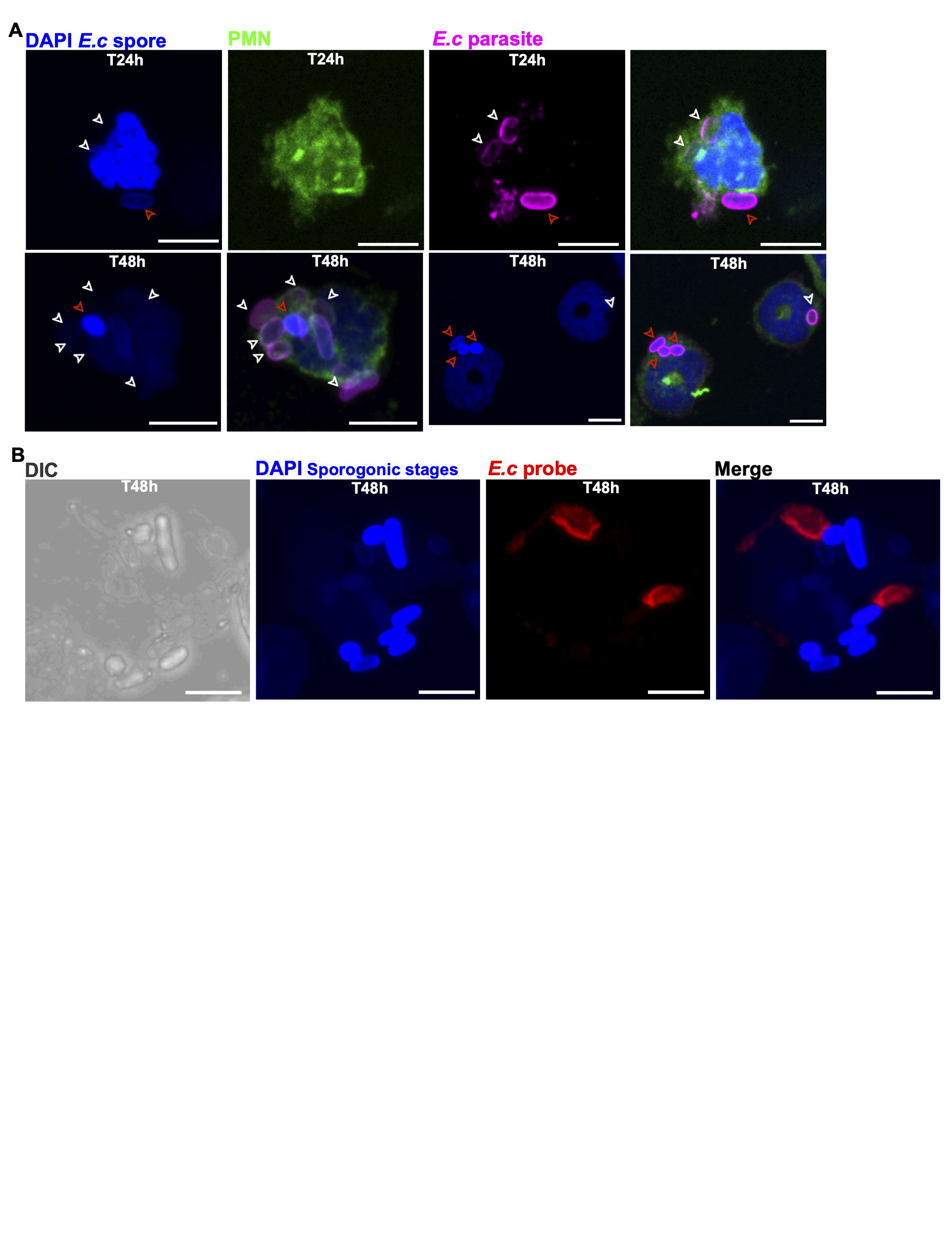

Supplement: Supplementary file 12 — Additional file 12: Figure S9. E. cuniculi parasites induce a recruitment of inflammatory cells in vivo in the mouse ear pinna model. [file 12866_2026_4989_MOESM12_ESM.tiff]

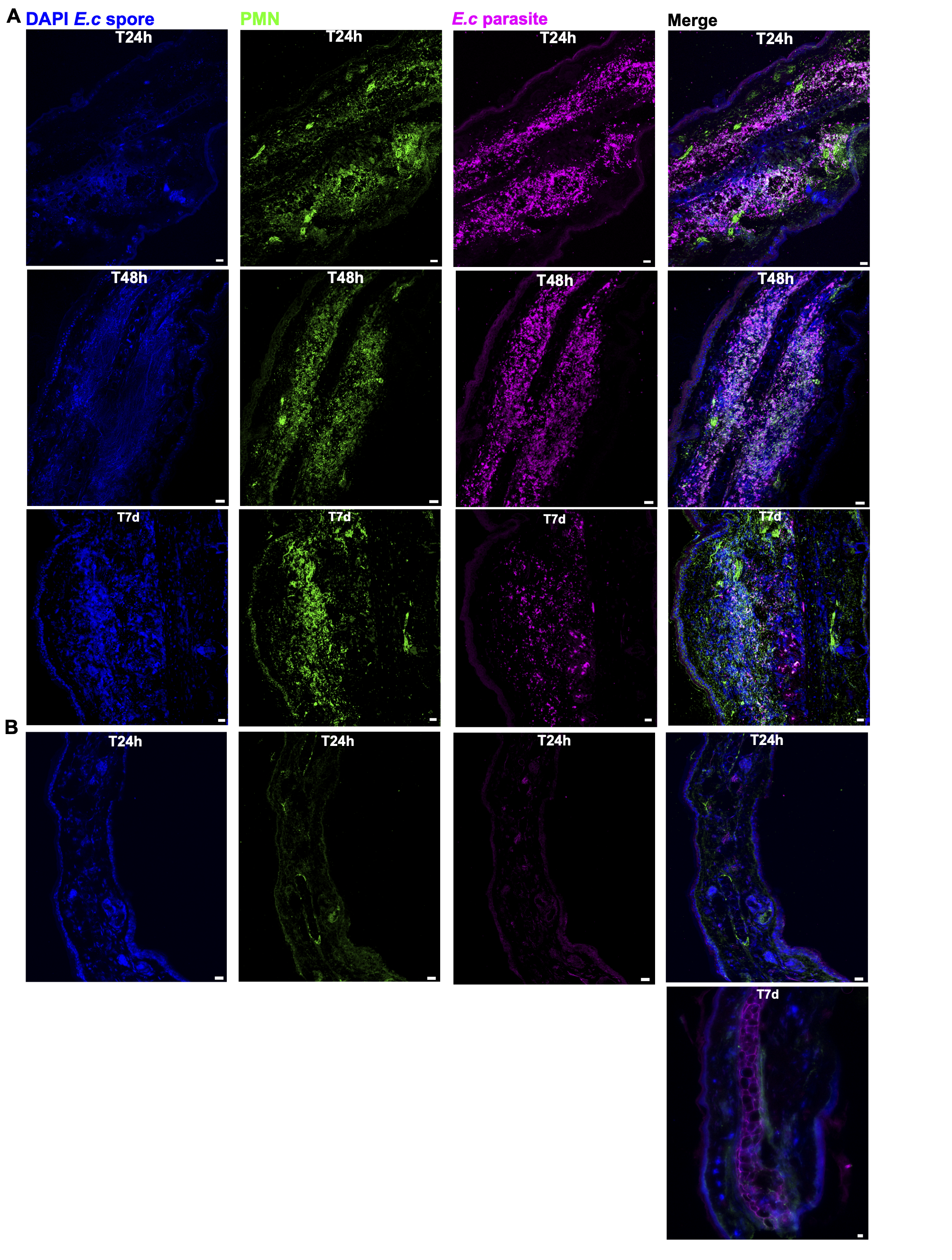

Supplement: Supplementary file 13 — Additional file 13: Figure S10. E. cuniculi parasites initiate development within PMNs in vivo. [file 12866_2026_4989_MOESM13_ESM.tiff]

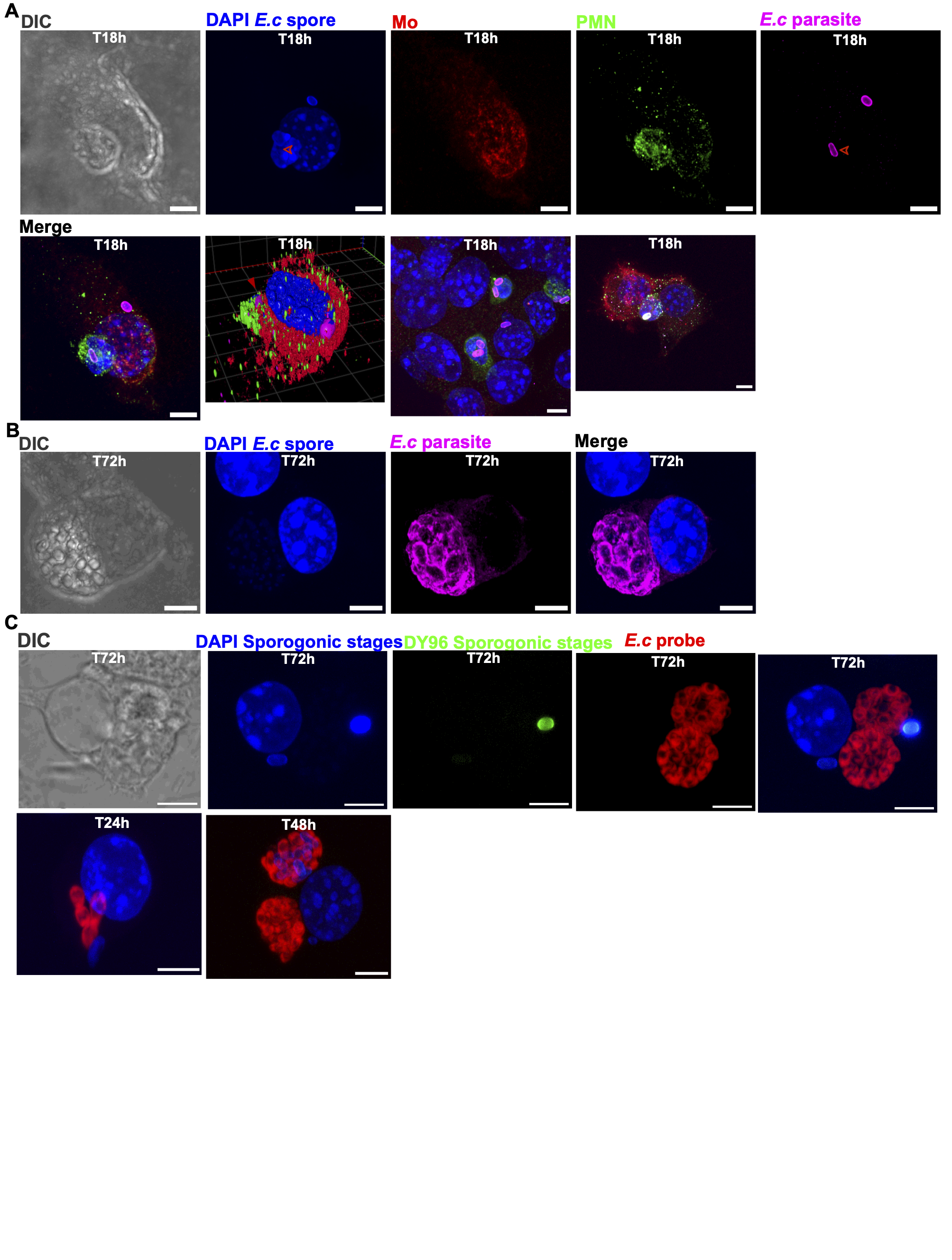

Supplement: Supplementary file 14 — Additional file 14: Figure S11. Entry of E. cuniculi parasites inside MOs Phagocytosis. [file 12866_2026_4989_MOESM14_ESM.tiff]

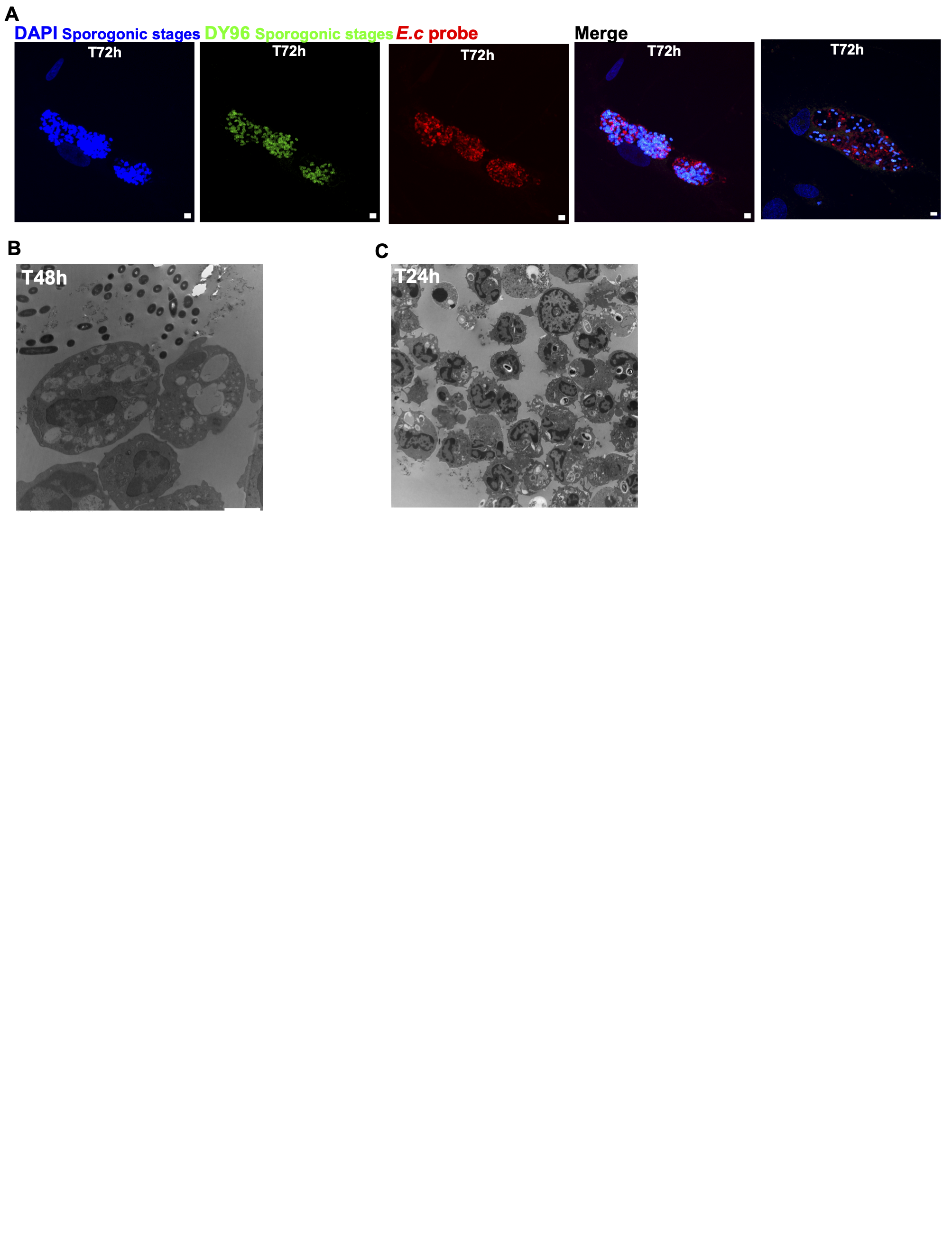

Supplement: Supplementary file 15 — Additional file 15: Figure S12. E. cuniculi development inside HFF cells after co-incubation with dying PMNs harboring intracellular parasites. [file 12866_2026_4989_MOESM15_ESM.tiff]
