# Supplementary material for: llluminating the live-cell dynamics of early interactions between neutrophils and the microsporidian parasite Encephalitozoon cuniculi
Source: BMC Microbiol. 2026 May 11;26:587. doi: 10.1186/s12866-026-04989-7 (PMC13330375; doi:10.1186/s12866-026-04989-7)
Supplement: Supplementary file 16 — Additional file 16: Supplementary Material and Methods. [file 12866_2026_4989_MOESM16_ESM.docx]

**Supplemental Material and methods**

**Scanning electron microscopy of *Encephalitozoon cuniculi* purified spores.**

Ten million *E. cuniculi* CW-labeled spores were deposited on 0,2 mm SEM Pores (DTM9305, Jeol filters) with a 34G needle fitted to a NanoFil Syringe. After absorption, spores were fixed for 12 h at 4°C in 0.2 M/sodium cacodylate buffer that contained 4% paraformaldehyde and 2.5% glutaraldehyde, at pH 7.4. They were then processed for SEM analysis as previously described (Carriere et al, 2023).

**Purification of murine PMNs**

Murine PMNs were isolated from the bone marrow of LysM-EGFP or C57Bl6 mice, as previously described (18–20). Femurs and tibias were removed, and heads of bones were cut off. Using a 26G syringe, bone marrow was flushed with buffer (PBS-0.5% BSA-EDTA 2 mM) and collected in Roswell Park Memorial Institute medium (RPMI) supplemented with 10% FCS, 1% L-glutamine, 15 mM HEPES, and 1 mM Pyruvate Sodium. Bone marrow was then passed through a 40 μm cell filter. After centrifugation (5 min at 500xg), red blood cells were lysed with 0.83% ammonium chloride for 10 min at RT. Bone marrow cells were then rinsed with buffer and centrifuged. PMNs were isolated from the bone marrow using a Neutrophil Isolation Kit (Miltenyi Biotec). The isolated PMNs were labeled with anti-CD11b (PE, Clone M1/70.15.11.5; dilution 1:100, Miltenyi Biotec) and anti-Ly6G (Anti-Ly-6G-APC-Vio770, REA526; 0.3 µg/mL, Miltenyi Biotec) antibodies for 25 min on ice. Sytox (0.5µM, Molecular Probes) was further added for 20 min to measure the viability of purified cells using an LSR FORTESSA X-2,0 (BD Biosciences). The gating strategy excluded cell debris and doublets.

**Immunolabelings of HFF, PMNs or J774 infected cells *in vitro***

HFF cells

A double immunolabeling was performed to quantify the proportion of adherent, intracellular, and developing parasites within HFF cells. The first step, without cell permeabilization, aimed to identify adherent parasites. After saturation with PBS-BSA 1%, a rabbit polyclonal anti-*E. cuniculi* serum (2 µg/mL) was applied for 2 h RT. Polyclonal anti-*E. cuniculi* antibodies correspond to sera from rabbits naturally infected with this parasite and were kindly provided by the company Hypharm SAS (France). After several washes with PBS-BSA 1%, a secondary anti-IgG AF 488 rabbit antibody (2 µg/mL, Thermo Fisher Scientific) was added for 1 h at RT. A second immunolabelling was then carried out after HFF cells membrane permeabilization, as follows. After a 1 h saturation step in PBS-BSA 1%, the cells were permeabilized with PBS-Triton X-100 0.5% (Sigma) for 20 min at RT. The polyclonal anti-*E. cuniculi* serum was then applied again (2 h at RT) (0.02 mg/mL). After several washes with PBS-Triton 0.1%, a secondary anti-IgG AF 647 rabbit antibody was added for 1 h at RT (0.02 mg/mL, Thermo Fisher Scientific). A DAPI (0.4 mg/L Sigma Aldrich) counterstaining was performed for 30 min at RT. Samples were mounted with Prolong Diamond AntiFade Mountant (Molecular Probes) and observed with a 40x objective on a ZEISS Axio Imager Apotome. Acquisition was done randomly using ZEN software. The proportions of adherent (red and green fluorescence), intracellular and developing parasites (only red fluorescence), were quantified. Around 10 to 12 fields were quantified per time point, for a total of 50 to 160 HFF cells.

PMNs

A specific immunolabeling was performed on collected PMNs to quantify the proportion of adherent and intracellular parasites. PMNs membranes were labeled using Cytopainter dye (Abcam) (100 μL (1X) for 2 x 10^6^ cells/mL). After 30 min at 37°C, cells were centrifuged and washed three times with buffer. The cells were then centrifuged using a CytoSpin centrifuge (Shandon) (12 x g, 5 min), and cell spots were fixed with 4% PFA for 5 min. Glass slides were stored at -20°C. To perform immunolabelling, PMNs were thawed at RT for 20 min, rehydrated with PBS for 20 min in a humid chamber, and then incubated with PBS-Triton 0.01%-BSA 1% for 1 h for the saturation step.

Immunolabelings were performed on J774 infected cells to analyze parasite development inside these cells. A detailed description of the immunolabeling protocol is given in supplementary Methods and Protocols section.

J774 cells

Coverslides of infected cells stored at 4°C were first washed three times with PBS and then incubated with PBS-Triton 0.01%-BSA 1% for 1 h for the saturation step. Immunolabelings were then performed on infected PMNs or J774 cells by applying specific antibodies for 2 h at RT. The primary antibody, a polyclonal antiserum obtained from an *E. cuniculi*-infected rabbit (2 μg/mL, PBS-BSA 1%-Triton 0,01%), was followed by an anti-rabbit IgG-AlexaFluor 488-labeled secondary antibody (2µg/mL Thermo Fisher Scientific, PBS-BSA 1%-Triton 0,01%) during 1h. The anti-*E. cuniculi* polyclonal serum detected all parasitic development stages. All samples were counterstained using DAPI (0.4mg/L Sigma Aldrich) and mounted with ProLong Diamond Antifade Mountant (Thermo Fisher Scientific).

**Transmission electron microscopy of infected PMNs**

At one- and two-days pi, PMNs were collected, and Ly6G^+^CW^+^ living infected PMNs were sorted using a FACS Aria Fusion SORP cell sorter. Sorted and unsorted cells were fixed overnight at +4°C in a solution containing 2.5% glutaraldehyde and 0.5% PFA in 0.2 M sodium cacodylate buffer (pH 7.4), and then washed in the same buffer. The pelleted cells were embedded in PBS-10% gelatin. The blocks were post-fixed for 1 h with 1% OsO_4_ in 0.2 M Na cacodylate buffer (pH 7.4) and washed three-times (10 min each) in the buffer. Dehydration was carried out in graded ethanol followed by acetone, and resin infiltration using an Automate Micro-Wave (Leica Microsystem). Specimens were embedded in resin overnight at RT and then cured for 2 days at 60°C**.** Thin sections (70 nm) were cut using a UC7 ultramicrotome (Leica Microsystem) and stained with uranyl acetate and lead citrate according to Reynolds. The sections were observed at 80 kV with a H-7650 transmission electron microscope (Hitachi) with an AMT40 camera (Hamamatsu). All chemical reagents were obtained from Electron Microscopy Science and distributed in France by Delta Microscopies.

**Immunolabelings of ear tissue cryosections from *E. cuniculi*-infected mice**

Immunolabeling was performed on ear tissue cryosections from infected mice, collected between 2 h and 7 days pi, or post-inoculation of PBS. A detailed description of the immunolabeling protocol is given in supplementary Methods and Protocols section. Cryosections performed as previously described (10) were thawed at RT for 30 min, rehydrated with PBS for an additional 30 min, and then incubated with a PBS-Triton 0.1%-BSA 2% solution for 1 h. The primary antibody anti-NIM-PR14: sc-59338 (1µg/mL), a marker of Ly6G+ PMN and Ly6C+ ^high^ inflammatory monocytes (Santa Cruz Biotechnology, Inc.) was applied overnight at 4°C, followed by an anti-rat IgG-AlexaFluor  546-labeled secondary antibody (2µg/mL Thermo Fisher Scientific, incubated for 1 h) to visualize PMNs; and a polyclonal *E. cuniculi*-antiserum (2 μg/mL) was also applied overnight at 4°C, followed by an anti-rabbit IgG-AlexaFluor  647-labeled secondary antibody (2µg/mL Thermo Fisher Scientific, incubated for 3h) to visualize parasites on the same cryosections. All samples were counterstained with DAPI (0.4mg/L, Sigma Aldrich) and mounted with ProLong Diamond Antifade Mountant (Thermo Fisher Scientific).

**Immunolabelings of PMNs purified from cutaneous *E. cuniculi*-infected tissues**

Specific immunolabelings were performed on PMNs purified from ear tissues of mice infected for 48 h to detect intracellular parasites. Frozen spots of purified PMNs were thawed at RT for 20 min, rehydrated with PBS for another 20 min in a humid chamber, and then incubated for 1 h with a PBS-BSA 1%-Triton 0.01% solution for saturation. An anti-NIMPR14 antibody (sc-59338) (Santa Cruz Biotechnology) was applied (1 μg/mL) in PBS-BSA 1%-Triton 0.01% for 2 h at RT, followed by an anti-rat-IgG-AlexaFluor 546-labeled secondary antibody (2 μg/mL for 1 h, Thermo Fisher Scientific) to visualize inflammatory cells as PMNs. To detect parasites, a polyclonal *E. cuniculi*-antiserum (2 μg/mL) was used, followed by an anti-rabbit IgG-AlexaFluor 488-labeled secondary antibody (2 μg/mL for 1 h, Thermo Fisher Scientific). All samples were counterstained with DAPI (0.4mg/L, Sigma Aldrich) and mounted with ProLong Diamond Antifade Mountant (Thermo Fisher Scientific).

**Imaging the parasite development using Fluorescence *in situ* hybridization**

FISH was performed on infected and uninfected PMNs purified from the bone marrow or from the ear tissue of infected mice, on J774 infected cells or on HFF cells co-incubated with *E. cuniculi*-infected sorted PMNs *in vitro*. Infected cell spots on glass slides were rehydrated in PBS at RT for 15 min, then incubated in a 1:1 solution of PBS and hybridization buffer (HB: 20 mM Tris–HCl pH 7.8, 0.9 M NaCl, 1X Denhardt’s solution, 0.01% SDS) for 15 min, followed by incubation in HB at 47°C for 20 min. A 5′-Cy3.5-labeled probe was added at a concentration of 0.5 μM, and hybridization was performed at 47°C for 2 h. Samples were subsequently washed in HB at 47°C for 30 min, followed by washing in HB at RT for 30 min, and finally in PBS at RT. Counterstaining was done overnight at RT using DAPI (0.4mg/L Sigma Aldrich), DY96 (1 μM, Sigma Aldrich), and 0.1% SDS (Sigma).

**Video microscopic imaging and data analysis**

Randomly acquired videos were analyzed using Imaris software to detect both cells and parasites. Parasites were detected using the CW signal as "spots" (diameter = 1.8 µm, quality=25), while PMNs were segmented based on the GFP signal (smoothing = 0.325 µm, background subtraction diameter = 6 µm, with manual thresholding set between 25 and 30 depending on image quality), and tracked as "surfaces" (autoregressive motion, 20 µm). PMNs located at the image border (distance < 3 µm) were excluded, and trajectories were manually corrected. A new channel containing the segmented PMNs labels was created from the Imaris "surfaces". These labels were dilated in Fiji using MorpholibJ, through a newly set up ImarisXT script, in order to determine, for each parasite in the vicinity of a cell, the identity of the cell concerned. Using Imaris software and displacement x/y data, cell morphology parameters (area, volume, and prolate ellipticity) were quantified, as well as cell movement over time (mean speed, displacement length, and trajectory straightness). For parasites, the shortest distance to the closest cell was calculated, and the corresponding cell ID was found using the dilated label. Quantitative data obtained were further analyzed using Excel software.

**Cytokine assay**

Collected samples intended for cytokine analysis were first thawed on ice and centrifuged for 10 minutes at 10,000 g at 4°C. Cytokines IL2, IL-4, IL-5, IL-6, IL-10, IL-12, GM-CSF, IFNγ and TNFα were quantified in the supernatants of PMNs cultures. A total protein assay was first performed on the supernatants of infected and non-infected PMNs cultures using the Bradford method, to estimate the total protein concentration. A standard curve was prepared from a mixture of purified cytokines through a series of serial dilutions. Cytokine quantification was carried out according to the manufacturer’s instructions, using the Bio-Rad Bio-Plex Pro Assays kit. Fluorescence intensity was measured with the Bio-Plex system to determine the concentrations of the cytokines of interest (pg/ml).
